# HCV replication in gastrointestinal mucosa: Potential extra-hepatic viral reservoir and possible role in HCV infection recurrence after liver transplantation

**DOI:** 10.1371/journal.pone.0181683

**Published:** 2017-07-27

**Authors:** Giovanna Russelli, Paola Pizzillo, Gioacchin Iannolo, Floriana Barbera, Fabio Tuzzolino, Rosa Liotta, Mario Traina, Giovanni Vizzini, Bruno Gridelli, Ester Badami, Pier Giulio Conaldi

**Affiliations:** 1 Department of Laboratory Medicine and Advanced Biotechnologies, IRCCS-ISMETT (Istituto Mediterraneo per i Trapianti e Terapie ad alta specializzazione), Palermo, Italy; 2 Vita-Salute San Raffaele University, Milan, Italy; 3 Office of Research, IRCCS-ISMETT, Palermo, Italy; 4 Pathology Service, Department of Diagnostic and Therapeutic Services, IRCCS-ISMETT, Palermo, Italy; 5 Endoscopy Service, Department of Diagnostic and Therapeutic Services, IRCCS-ISMETT, Palermo, Italy; 6 Department for the Treatment and Study of Abdominal Diseases and Abdominal Transplantation, IRCCS-ISMETT, Palermo, Italy; 7 Fondazione Ri.MED, Palermo, Italy; Inserm U0152, UMR 5286, FRANCE

## Abstract

**Purpose:**

Hepatitis C virus (HCV) predominantly infects hepatocytes, although it is known that receptors for viral entry are distributed on a wide array of target cells. Chronic HCV infection is indeed characterized by multiple non-liver manifestations, suggesting a more complex HCV tropism extended to extrahepatic tissues and remains to be fully elucidated. In this study, we investigated the gastrointestinal mucosa (GIM) as a potential extrahepatic viral replication site and its contribution to HCV recurrence.

**Methods:**

We analyzed GIM biopsies from a cohort of 76 patients, 11 of which were HCV-negative and 65 HCV-positive. Of these, 54 biopsies were from liver-transplanted patients. In 29 cases, we were able to investigate gastrointestinal biopsies from the same patient before and after transplant. To evaluate the presence of HCV, we looked for viral antigens and genome RNA, whilst to assess viral replicative activity, we searched for the replicative intermediate minus-strand RNA. We studied the genetic diversity and the phylogenetic relationship of HCV quasispecies from plasma, liver and gastrointestinal mucosa of HCV-liver-transplanted patients in order to assess HCV compartmentalization and possible contribution of gastrointestinal variants to liver re-infection after transplantation.

**Results:**

Here we show that HCV infects and replicates in the cells of the GIM and that the favorite hosts were mostly enteroendocrine cells. Interestingly, we observed compartmentalization of the HCV quasispecies present in the gastrointestinal mucosa compared to other tissues of the same patient. Moreover, the phylogenetic analysis revealed a high similarity between HCV variants detected in gastrointestinal mucosa and those present in the re-infected graft.

**Conclusions:**

Our results demonstrated that the gastrointestinal mucosa might be considered as an extrahepatic reservoir of HCV and that could contribute to viral recurrence. Moreover, the finding that HCV infects and replicates in neuroendocrine cells opens new perspectives on the role of these cells in the natural history of HCV infection.

## Introduction

Hepatitis C virus (HCV) belongs to the virus family of *Flaviviridae*, whose members are known to infect a wide array of target cells, resulting in multi-faceted disease expression. However, HCV predominantly infects hepatocytes, leading to acute and chronic hepatitis.

HCV particles are constituted by a single-stranded positive RNA genome, the Core antigen and the envelope glycoproteins, E1 and E2. The viral envelope is a lipid membrane in which the glycoproteins E1/E2 are anchored. This structure coats the nucleocapsid, consisting of the core protein and HCV genome. Envelope glycoproteins mediate viral entry in the host cell, having a role in receptor binding and membrane fusion between viral envelope and the host cell. HCV entry in the host cell requires a cascade of coordinated and consecutively ordered events where virus binds to a number of receptors. They include SRB1 [1], tetraspanin CD81 [2], tight-junction proteins claudin-1 [3] (CLDN1) and occludin (OCLN) [4] and the LDL receptor (LDLR) [5] and are expressed by many cell types. HCV viral particles have indeed been found in peripheral mononuclear cells (PBMC) for instance, as well as cells of the spleen, intestine, pancreas, heart, kidneys, brain, lymph nodes, dendritic cells, B and T lymphocytes [6–14]. Besides hepatitis, HCV chronic infection is indeed characterized by several extrahepatic manifestations including hematologic diseases such as cryoglobulinemia and lymphoma, autoimmune disorders such as thyroiditis, renal disease or dermatologic syndromes such porphyria cutanea, cardiovascular diseases, metabolic syndromes like diabetes and nervous system illnesses [15]. This wide array of non-liver diseases implies a more complicated and knitted HCV tropism, extended to extrahepatic tissues and remains to be fully elucidated.

20–30% of HCV infected patients spontaneously eradicate infection by mounting humoral and cellular immune response [16–19]. However, in the remainder population, HCV establishes chronic infection aided by the high replicative rate, the increasing genetic variability and the ability to evade immunological pressure [20–27]. The discovery and clinical use of new generation direct-acting antivirals (DAAs) has signed an invaluable milestone in the treatment of chronic HCV infection. The use of the nucleotide polymerase inhibitor Sofosbuvir, the protease inhibitor Simeprevir and HCV NS5A inhibitor Ledipasvir and other drugs introduced more recently in different combinations has allowed the achievement of sustained virological response (SVR) rates higher than 90% in patients with chronic hepatitis in an interferon-free regimen. The current European recommendations on HCV treatment have broaden the range of treatable patients, including those categories that were previously excluded, such as heavily decompensated cirrhotic, patients with renal failure or high MELD score [28]. Recently, DAAs therapies have shown to provide benefit also on extrahepatic manifestations in treated patients [29]. However, data on possibly upcoming long-term side effects of DAAs therapies are not available yet, involving emergence of drug Resistant Associated Substitutions [30] or extrahepatic side effects, for instance. For example, the detrimental effects of anti-viral therapies on liver tumor recurrence in patients that have developed HCV-related hepatocarcinoma has been reported [31]. Results from clinical trials on the treatment of liver transplanted HCV patients are only recently emerging, showing that SVR rates are less satisfactory than expected, scoring an average of 70% [32]. Recently, a study conducted in a cohort of HCV patients treated with DAAs after liver transplantation has revealed the existence of occult HCV infection in PBMC and liver, implying responsibility for liver reinfection [33]. In some cases, treatment for HCV related end-stage liver disease remains orthotopic liver transplantation (OLT) [19, 24, 34]. After transplantation, however, the newly engrafted liver is universally re-infected, likely due to the “residual” virus still circulating systemically and/or vehicled from extrahepatic reservoirs [35, 36]. In our study, we investigated the role of the gastrointestinal mucosa (GIM) as an extrahepatic reservoir, addressing its contribution to post-transplant liver re-infection. To sustain productive HCV infection, permissive cells must allow viral entry and sustain viral replication and infection transmission via release of infectious viruses and/or cell-to-cell contact. Here, we investigated if GIM cells sustain HCV replication acting as productive extrahepatic virus reservoir and playing a role in the natural history of HCV infection and persistence. We hypothesized that HCV-positive gastrointestinal epithelial cells could produce and release in the blood HCV particles able to infect the liver via the enterohepatic circulation. To test this hypothesis we analyzed GIM biopsies from HCV+ patients. To assess HCV infection, we determined the presence of viral proteins in the GIM biopsies before and after OLT by immunohistochemistry and quantification of total HCV RNA. By co-localization imaging, we identified the specific cell types preferentially infected by the virus. Furthermore, we determined the presence of replicating virus by minus-strand HCV RNA detection, representing a replicating intermediate. Finally, we studied HCV compartmentalization through quasispecies analysis, in order to underpin the contribution of GIM viral variants to liver re-infection.

## Results

### Gut biopsies of HCV patients are positive for viral RNA and proteins

We studied GIM biopsies from a cohort of patients referred to Ismett from 2005 to 2014. In particular, we analyzed GIM biopsies form 76 patients, 11 of which were HCV-negative and 65 HCV-positive. Of these, 54 biopsies were from liver-transplanted patients. In 29/54 cases, we were able to investigate gastrointestinal biopsies from the same patient before and after OLT. To increase assay sensitivity, detection of viral RNA was performed by nested-PCR. We found that 79% of GIM biopsies collected before transplantation and 69% collected after transplantation were positive for HCV RNA. To determine if the detected viral RNA was derived from HCV actively replicating in GI tissues, we searched for the presence of minus-strand HCV RNA in the bioptic samples. 73% of HCV RNA+ biopsies before OLT and 40% of HCV RNA+ biopsies after OLT were also positive for virus minus RNA strand, indicating HCV active replication (Table 1). Moreover we found HCV RNA in 36% of the biopsies from the remainder 25/54 HCV transplanted patients and 89% of them were also positive for HCV RNA minus strand. In all cases we found HCV RNA in GIMs biopsies of HCV-infected patients, independently to HCV viremia positivity. These data show that HCV RNA minus strand appears to be significantly prevalent in GI tissues of HCV-infected patients (p<0.05). Moreover, since the negative strand is approximately 10% of the positive strand concentration in HCV infected cell cultures [37], false negatives might result, at least in part, due to technical limitations as only small amounts of good quality RNA are extractable from formalin fixed paraffin-embedded samples. Although most of the analyzed tissues were of gastric origin, we did not notice any difference in the presence of HCV RNA and minus strand between biopsies from stomach and intestine.

**Table 1:** Demographic, clinical and laboratory data of HCV liver-transplanted patients.

Patient N°	Age	Gender	HCV GT	HCV RNAemia (IU/ml)		Pre-OLT GIM biopsy			Post-OLT GIM biopsy		
				Pre-OLT (months pre-OLT)	Post-OLT (months post-OLT)	Months	HCV RNA/ HCV minus	IHC	Months	HCV RNA/ HCV minus	IHC
1	70	M	1b	3.6x10^6^ (1)	9.35x10^5^ (16)	1	+/-	+	15	+/-	+
2	57	M	1b	1.2x10^5^ (1)	9.50x10^6^ (7)	1	+/-	-	16	+/-	+
3	57	M	1b	1x10^5^ (18)	5.00x10^6^ (1)	18	+/+	+	0	+/-	+
4	65	F	1b	ND	1.73x10^7^ (7)	1	-/-	+	7	+/-	+
5	56	M	1b	ND	9.27x10^5^ (9)	14	+/+	+	0	+/-	+
6	72	F	1b	2x10^5^ (1)	1.84x10^7^ (71)	1	+/+	-	2	-/-	-
7	72	M	1b	>7x10^5^ (4)*	8.74x10^6^ (44)	4	+/+	+	42	+/+	+
8	68	F	1b	>7x10^5^ (22)*	3.48x10^6^ (17)	22	+/+	+	17	+/+	+
9	66	M	1b	1.2x10^5^ (11)	2.75x10^7^ (13)	12	+/+	+	13	+/-	+
10	54	M	2a/2c	9.7x10^5^ (0)	8.9x10^6^ (15)	5	+/+	+	4	-/-	+
11	69	M	1b	>7x10^5^ (6)*	1.49x10^6^ (49)	12	-/-	+	42	+/+	+
12	50	M	1b	>7x10^5^ (4)*	2.89x10^5^ (62)	7	+/+	+	59	-/-	+
13	64	M	1b	>7x10^5^ (4)*	1.47x10^7^ (47)	19	-/-	+	2	-/-	+
14	70	M	1b	>7x10^5^ (2)*	1.03x10^5^ (33)	6	+/+	+	34	+/+	+
15	64	M	1b	>7x10^5^ (4)*	7.08x10^6^ (40)	6	+/+	+	37	-/-	+
16	69	M	1b	>7x10^5^ (10)*	1.50x10^6^ (22)	10	+/+	+	3	-/-	+
17	69	M	1b	>7x10^5^ (17)*	1.00x10^7^ (28)	17	-/-	+	28	-/-	+
18	69	M	1b	ND	2.98x10^6^ (41)	8	+/+	+	43	+/-	+
19	22	M	1b	Neg (30)	Neg (48)	4	+/-	+	17	+/-	+
20	54	M	1b	>7x10^5^ (24)*	8.47x10^5^ (18)	23	+/-	+	11	+/-	+
21	70	M	1b	1x10^9^ (2)	1.78x10^7^ (2)	1	-/-	+	2	-/-	+
22	48	M	3a	ND	6.00x10^6^ (12)	33	+/+	+	31	+/-	+
23	69	M	1b	>7x10^5^ (20)*	8.42x10^5^ (48)	19	+/-	+	48	+/+	+
24	66	M	1b	ND	1.27x10^6^ (19)	13	+/-	-	19	-/-	+
25	55	M	1b	>7x10^5^ (24)*	5.36x10^5^ (3)	0	+/+	+	9	+/-	+
26	59	F	1b	ND	1.76x10^6^ (14)	6	+/+	+	21	+/-	+
27	62	F	1b	ND	4.30x10^3^ (0)	22	+/+	+	2	+/+	+
28	64	M	1b	4.2x10^5^ (2)	4.27x10^5^ (2)	4	+/+	+	14	+/+	+
29	65	M	1b	>7x10^5^ (31)*	ND	31	-/-	+	1	+/+	+
50	62	M	3a		3.93x10^5^ (13)				13	+/+	+
51	59	M	1b	5.5x10^4^ (31)		31	+/+	+			
52	67	M	1b		2.33x10^6^ (104)				104	-/-	+
53	64	M	1b		3.28x10^5^ (37)				39	-/-	+
54	68	M	1b		3.22x10^6^ (0)				0	-/-	-
55	73	M	1b		2.67x10^5^ (88)				92	-/-	+
56	51	M	3a		1.11x10^6^ (32)				32	-/-	+
57	58	M	1b		9.86x10^6^ (23)				19	-/-	-
58	74	M	1b		1.31x10^6^ (17)				17	-/-	+
59	65	F	1b		9.09x10^2^ (9)				0	-/-	+
31	68	F	1b		5.26x10^5^ (26)				23	+/+	+
32	71	M	1b		3.81x10^5^ (1)				1	+/-	+
33	69	F	1b		3.51x10^6^ (72)				80	+/+	+
36	65	M	1b		8.53x10^6^ (4)				0	+/+	-
37	75	F	1b		1.09x10^6^ (113)				107	+/-	+
41	65	F	1b	1.1x10^6^ (0)		10	-/-	+			
42	56	M	3a		8.45x10^6^ (2)				7	-/-	+
44	70	F	1b		1.37x10^6^ (73)				13	+/+	+
45	63	M	1b	3x10^5^ (6)		45	+/+	+			
46	69	F	1b		2.37x10^6^ (61)				50	-/-	+
47	53	M	3a	2.9x10^6^ (4)		5	-/-	+			
49	60	F	1b	2.7x10^6^ (1)		4	-/-	+			
30	60	M	3a	2.3x10^7^		13	-/-	-			
43	70	M	1b	6.2x10^5^ (29)		15	-/-	+			
48	67	M	1b	6.3x10^3^ (0)		4	-/-	+			

ND: not done;

* samples analysed using COBAS Amplicor HCV Monitor test v.2.0, in COBAS Amplicor Analyzer (Roche Diagnostic).

**Fig 1:**
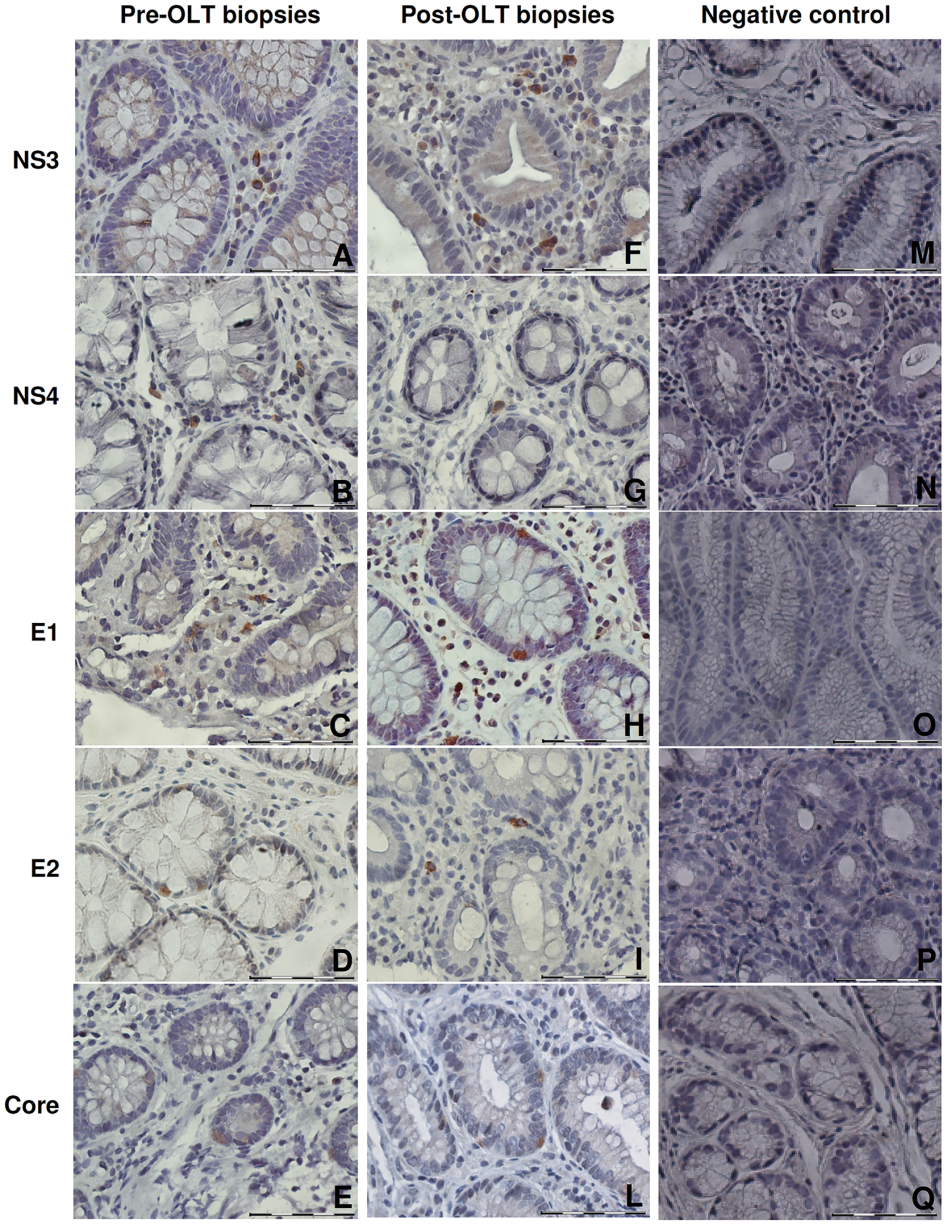
Immune-histochemical staining of HCV proteins on paraffin-embedded GIM biopsies sections of HCV-transplanted patients. Immune-histochemical staining of HCV proteins was performed on paraffin-embedded GIM biopsies sections of HCV-transplanted patients before (A-E) and after transplantation (F-L (Original magnification X400). (A, F) NS3 in stromal cells of duodenum polyps, (A patient 18, F patient 14); (B, G) NS4 in stromal cells of colon polyp (B patient 7, G patient 25); (C) E1 in stromal cells of colon (patient 18); (H) E1 in glandular epithelial cells of duodenum (patient 10); (D) E2 in glandular epithelial cells of rectum polyp (patient 9); (I) E2 in stromal cells of duodenum polyp (patient 14); (E) Core in glandular epithelial cells of duodenum (patient 15); (L) Core in glandular epithelial cells of cecum polyp (patient 10). Negative controls (M-Q) were performed by omitting the primary antibodies, and by using anti-mouse (M-O) and anti-goat (P, Q) secondary antibodies. (M) NS3 in antrum; (N) NS4 in polyp body; (O) E1 in antrum; (P) E2 in polyp colon; (Q) Core in polyp colon. Scale bar 50 μm.

**Fig 2:**
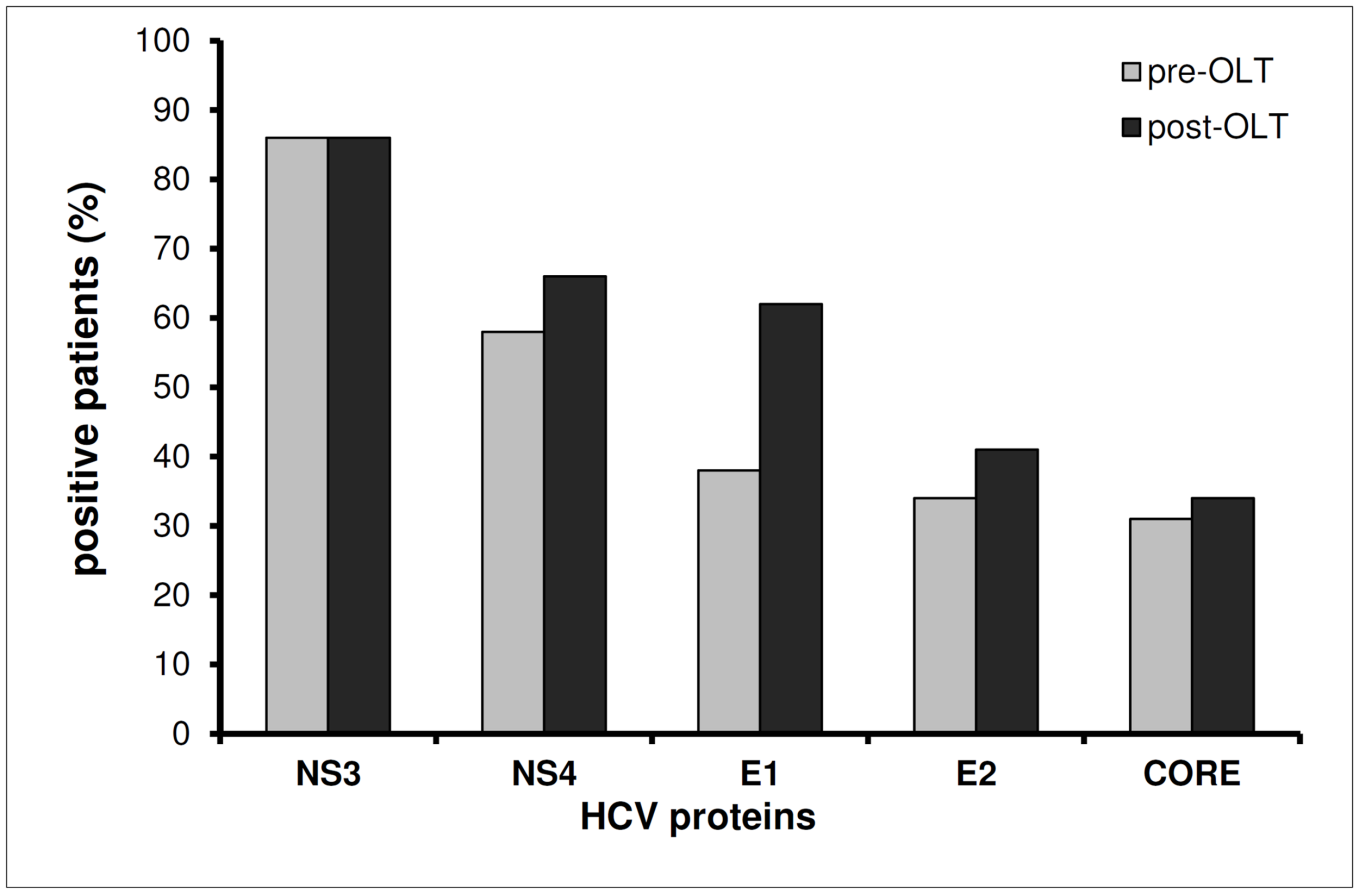
HCV proteins expression in pre- and post-transplant GIM biopsies of HCV-liver transplanted patients. Gut sections obtained pre- and post-OLT were analyzed for expression of HCV proteins NS3, NS4, E1, E2 and Core. Histograms represent the percentage of patients positive for the viral antigens described in the x axis. The analysis reveals no significant differences in the expression level of viral antigens before and after transplantation (p>0.05).

Data shown thus far were obtained from transplanted patients administered with immunosuppressive therapy. We wanted to assess HCV infectivity and replication rate also in immunocompetent host cells. To do this, we analyzed GIM biopsies from not-transplanted HCV positive patients. We had access to 11 GIM samples from not-transplanted HCV patients (Table 2). We found HCV RNA in 36% of biopsies of HCV positive patients, 75% of which were also positive for HCV RNA minus strand. By immunohistochemistry, the analyzed HCV+ GIM biopsies resulted to be positive both for structural (C, E1, E2 Ags) and non-structural (NS3, NS4) HCV proteins “Fig 1” confirming that the virus could actively replicate in GI cells. Morphological analysis of histological sections revealed that HCV proteins were mostly found in stromal cells and in enteroendocrine cells. However, as shown in “Fig 1”, only few cells resulted to be positive for HCV proteins in GIM tissues, except for some isolated samples as in “Fig 1F”. The analysis of the biopsies taken from the same patients showed that level of positivity for HCV proteins in GI cells is quite similar pre- and post-transplantation “Fig 2”. In particular, biopsies of 2 patients before OLT and 4 patients after OLT were positive for 5 HCV Ags; biopsies from 6 patients before and after OLT were positive for 4 Ags; biopsies from 8 patients before OLT and 5 patients after OLT were positive for 3 Ags; biopsies of 5 patients before OLT and 11 patients after OLT were positive for 2 Ags; biopsies from 5 patients before OLT and 2 patients after OLT were positive for 1 HCV Ag.

**Table 2:** Demographic, clinical and laboratory data of HCV positive not-transplanted patients.

patients N°	Age	Gender	HCV genotype	RNAemia	GIM biopsies	
					HCV RNA/ HCV minus	IHC
66	28	M	1b	5.5x10^1^	-/-	+
67	63	M	ND	2.0x10^2^	+/+	-
69	55	F	1b	4.6x10^4^	-/-	+
60	62	F	2a/2c	2.4x10^3^	+/+	-
61	57	M	2a/2c	7.7x10^5^	-/-	-
62	72	F	ND	1x10^6^	-/-	+
38	53	M	1b	5.2x10^5^	+/+	+
39	63	M	1b	4.1x10^5^	+/-	+
40	52	M	1b	4x10^6^	-/-	+
63	64	M	1b	4.8x10^3^	-/-	+
64	66	M	ND	6.1x10^5^	-/-	+

ND: not done.

### Detection of HCV receptors in GIM compartment

**Fig 3:**
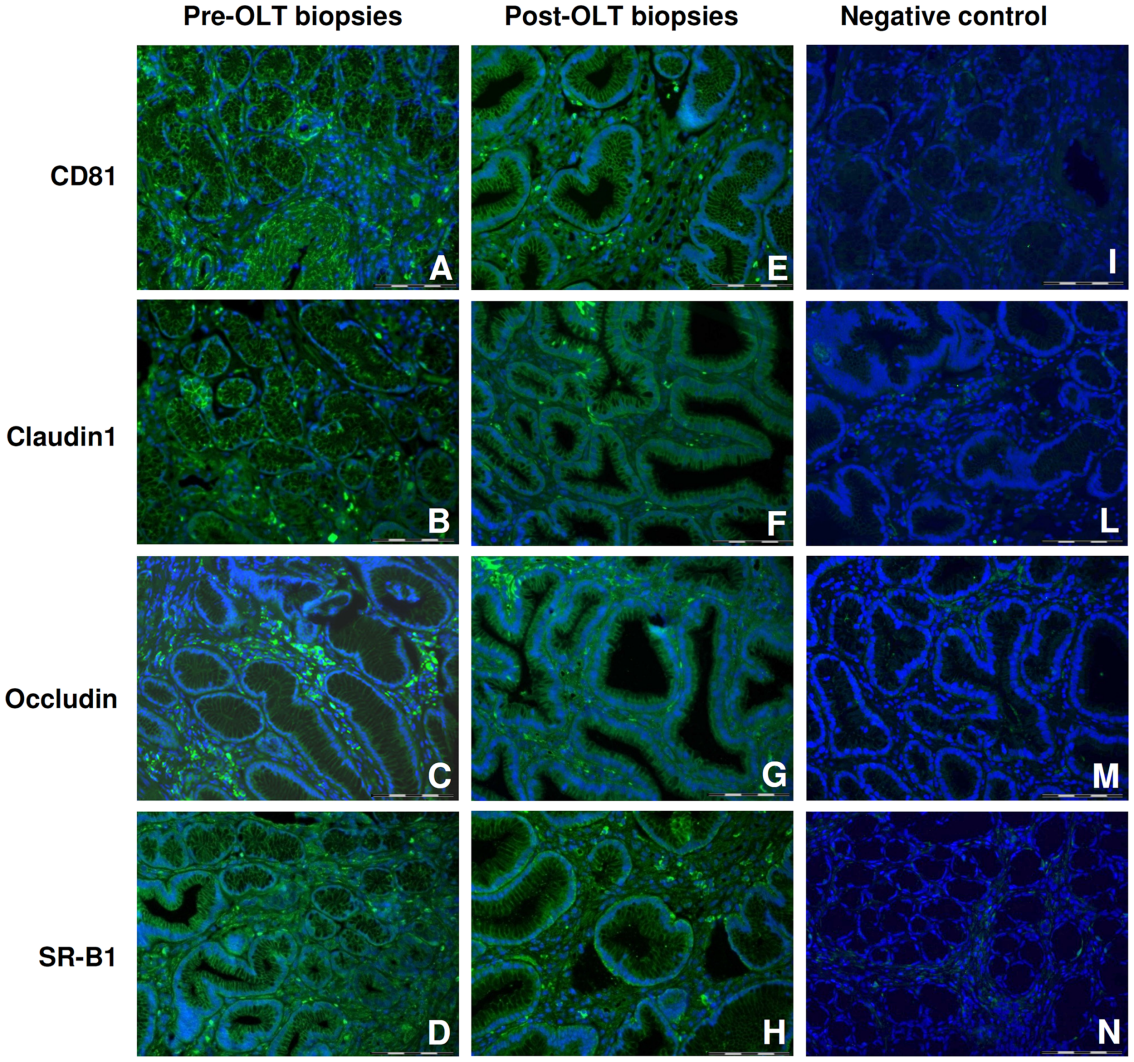
Immunofluorescence assay on GIM biopsies of HCV-transplanted patients. Immunofluorescence assay on GIM polyp biopsies of HCV-transplanted patients, using antibodies against HCV receptors: CD81, SR-B1, Claudin-1, Occludin (Original magnification X400). (A, E) CD81 in colon (patient 26); (B, F) Claudin-1 in colon (patient 26); (C, G) Occludin in colon (C patient 28, G patient 26); (D) SR-B1 in antrum (patient 28); (H) SR-B1 in colon (patient 26). All the sections were clearly positive for the analyzed HCV receptors before (A-D) and after transplantation (E-H). Negative controls (I-N) were performed on GIM biopsies of HCV positive patients by omitting the primary antibodies, and by using polyclonal FITC-conjugated Donkey anti-mouse (I) and anti-rabbit (L, M, N) as secondary antibodies. (I CD81 in antrum; L Claudin-1 in polyp colon; M Occludin in antrum; N SR-B1 in polyp colon. Nuclei were counterstained with DAPI (blue). Scale bar 50 μm.

HCV entry in the target cell is mediated by the mutual interaction between viral surface proteins and specific host receptors. To assess permissiveness to HCV infection of gastrointestinal cells, we tested, by immunofluorescence, the expression of known HCV ligands CD81, SR-BI, CLDN1 and OCLN in pre-OLT “Fig 3A, 3B, 3C and 3D” and post-OLT “Fig 3E, 3F, 3G and 3H” in histology sections of GIM biopsies from HCV patients. Biopsies showed a clear positivity for all the HCV receptors tested. The results also demonstrated that the expression pattern of HCV receptors in the GIM did not differ after transplantation. Also GIM biopsies from HCV-negative patients stained positively for HCV receptors, suggesting that the gastrointestinal mucosa is permissive to HCV infection “S1 Fig”.

### Detection of HCV core Ag and chromogranin A in neuroendocrine cells of GIM compartment

**Fig 4:**
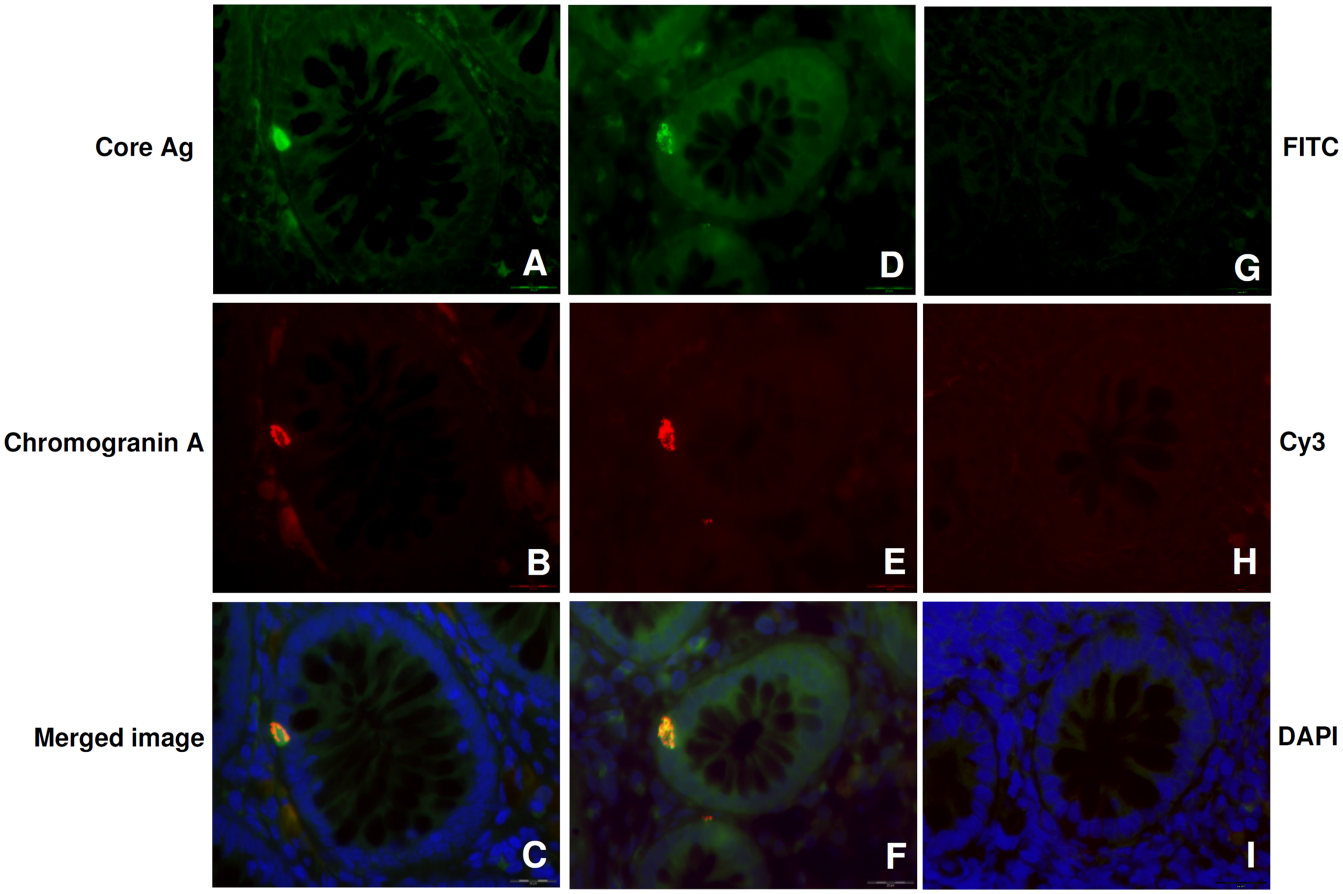
Immunofluorescence double staining assay on GIM biopsies of HCV-transplanted patients. Immunofluorescence assay on colon polyps of HCV-transplanted patients (*n* = 2), using antibodies against HCV core antigen (green) (A, D) and Chromogranin A (red) (B, E). Negative control was performed by omitting the primary antibodies and using polyclonal FITC-conjugated Donkey anti-goat (G) and Cy3-conjugated Donkey anti-mouse (H) as secondary antibodies. I, DAPI only. Nuclei were counterstained using DAPI. Scale bar: 20 μm. Double staining showed a clear positivity of enteroendocrine cells for HCV core Ag (merged C, F).

The intestine is the largest endocrine organ in the body where enteroendocrine cells in particular are widely distributed throughout the gastrointestinal tract [38]. Enteroendocrine cells are subcategorized into: enterochromaffin cells for the 70% that mainly secrete Chromogranin A, serotonin and synaptophysin; D cells that secrete somatostatin and L cells which produce proglucagon-derived peptides and peptide YY [39]. By immunohistochemistry we observed that cells that stained positively for HCV antigens were characterized by the morphology of stromal and neuroendocrine cells “Fig 1”. To confirm this observation and thus precisely identify the specific cell type infected by the virus, we performed an immunofluorescent co-staining on GIM sections, using antibodies against HCV core Ag (green) and chromogranin A (red), markers of enterochromaffin cells [39]. The results showed in “Fig 4” demonstrate that Chromogranin A and HCV Core Ag signals co-localize (merged image), thus confirming that enteroendocrine cells are permissive to HCV infection.

### Gene expression analysis of somatostatin genes in GIM

**Fig 5:**
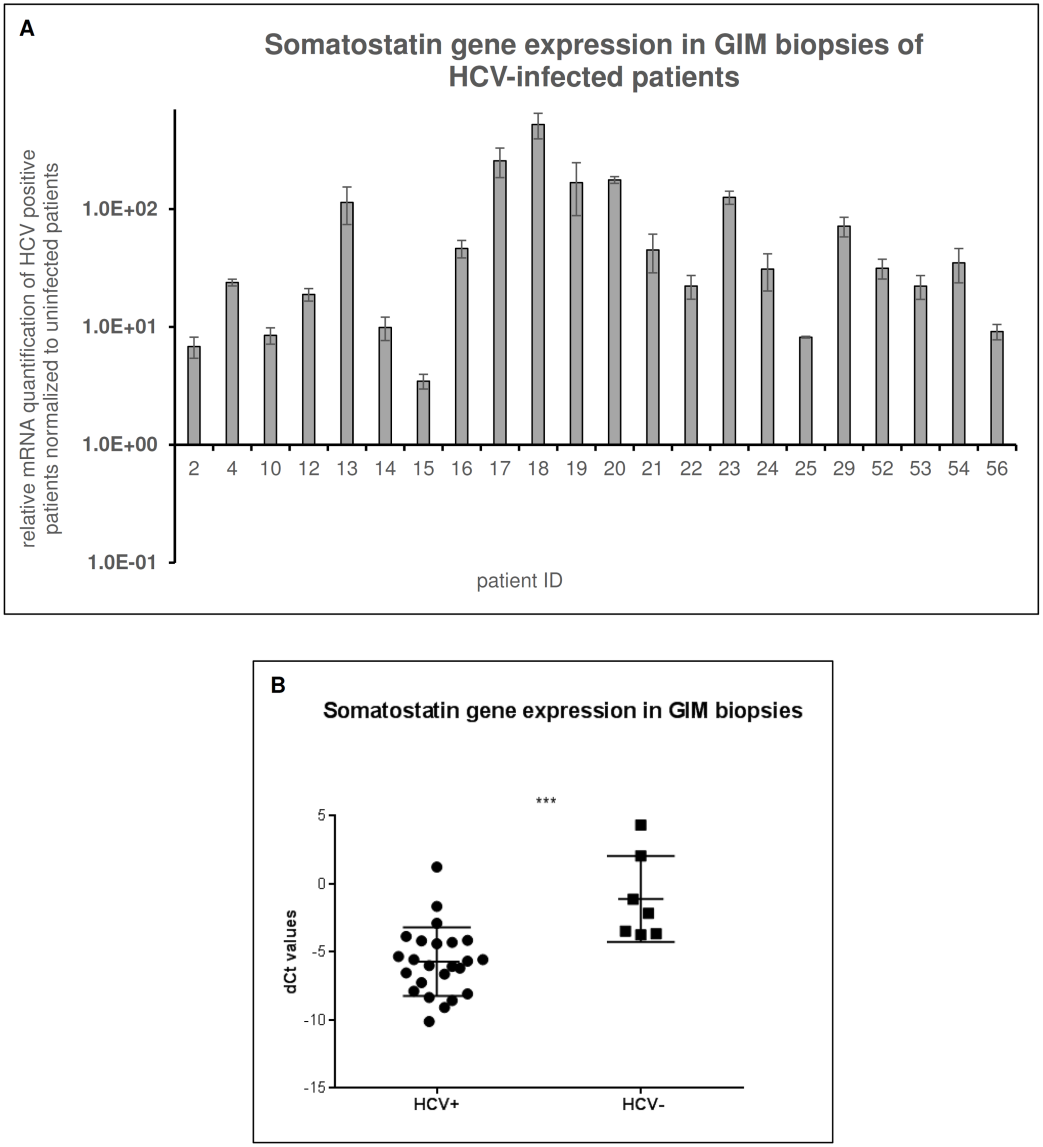
Semi-quantitative Real time PCR of Somatostatin gene in GIM biopsies of HCV-infected patients. Semi-quantitative Real time PCR of Somatostatin gene was performed in GIM biopsies of HCV-positive patients. All samples (*n* = 22) were performed in triplicate and normalized versus uninfected samples (*n* = 7). The analysis showed high gene expression levels in all patients (A), and the ΔCt values of HCV-positive patients resulted significantly higher than uninfected patients ΔCt (p<0.001) (B).

In order to underpin the ability of the virus to affect the function of enteroendocrine cells, we evaluated the expression levels of somatostatin, main gene marker of D cells function [39], in bioptic samples from 29 patients (HCV-positive *n* = 22; HCV-negative *n* = 7 as control). The results showed high levels of expression for somatostatin gene in all HCV patients normalized to uninfected controls “Fig 5A”. The comparison of the ΔCt values obtained from the HCV+ patients group and from the HCV- patients group showed a significant difference in gene expression (p<0.001) “Fig 5B”. This strongly suggests that virus affects the function of host cells by inducing significantly increased expression of the biomarker Somatostatin.

### NCI-H716 infection assay

**Fig 6:**
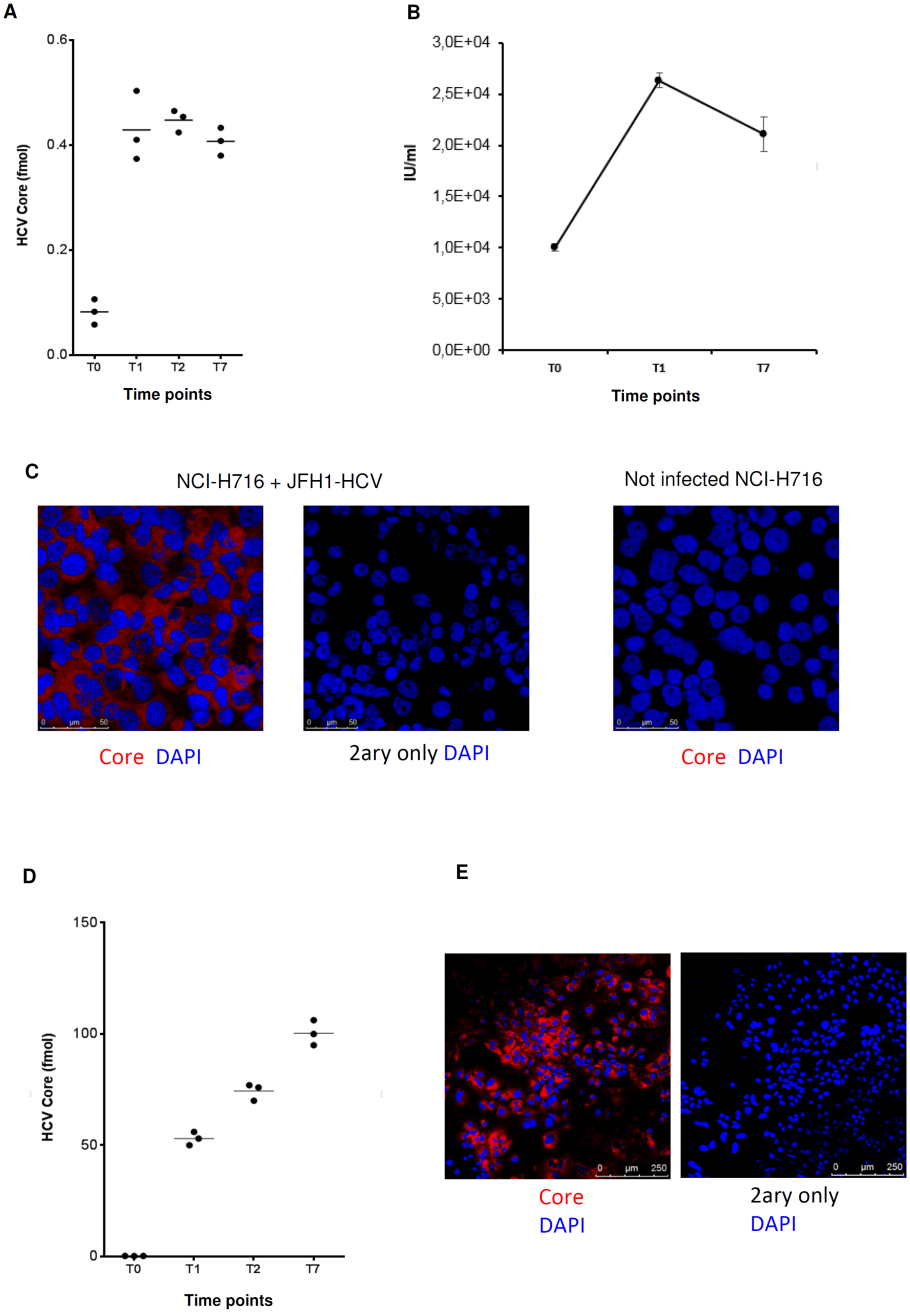
NCI-H716 and Huh 7.5 cells infection using HCV replicons. NCI-H716 cell line was infected *in vitro* using JFH1-HCV replicons and the infection levels were evaluated for 7 days after infection. Uninfected cells were used as negative control and Huh7.5 cells were infected using JFH1 as positive control. In both cell lines the HCV core titer in the supernatant of the infected cells was evaluated by Chemiluminescent Microparticle Immunoassay (CMIA) (A, D) and immunofluorescence assay (IF) was performed to stain the HCV Core protein (red) (C, E). Moreover, Real-time PCR was evaluated in NCI-H716 cell line to quantify the HCV viral load (B). For the IF assay, (C, E) nuclei were counterstained with DAPI (blue) and specificity of staining is demonstrated by the negative control with omission of primary antibody (C: scale bar 50μm; E: scale bar 250μm).

To confirm the ability of the virus to entry and replicate in neuroendocrine cells, we infected the NCI-H716 cells line with JFH1-HCV virions [40] produced in cell culture as described in material and methods [41]. NCI-H716 is a colorectal adenocarcinoma cell line usually used as a model of endocrine differentiation of intestinal epithelium [42]. Experiment was done in triplicate and viral replication serially assayed between days T0 and T7 after infection. In particular, we evaluated the infectious titer by quantifying the concentration of HCV Core Ag released in the culture medium by chemiluminescence “Fig 6A” and real-time PCR “Fig 6B”. Baseline viral titer was initially quite low as expected (T0) and then increased and remained stable between days 1 and 7 post-infection “Fig 6A and 6B”. In addition, as readout of infection we used immunofluorescence staining for HCV Core at T7 (red, “Fig 6C”) further demonstrating that NCI.H716 cells sustain viral infection. Nuclei were counterstained with DAPI (Blue, “Fig 6C”). As positive control, we used Huh-7.5 cells [43] “Fig 6D and 6E”. These data show that NCI-H716 cells are permissive to HCV infection and sustain active replication.

### Gene expression analysis of interferon-stimulated genes in GIM

**Fig 7:**
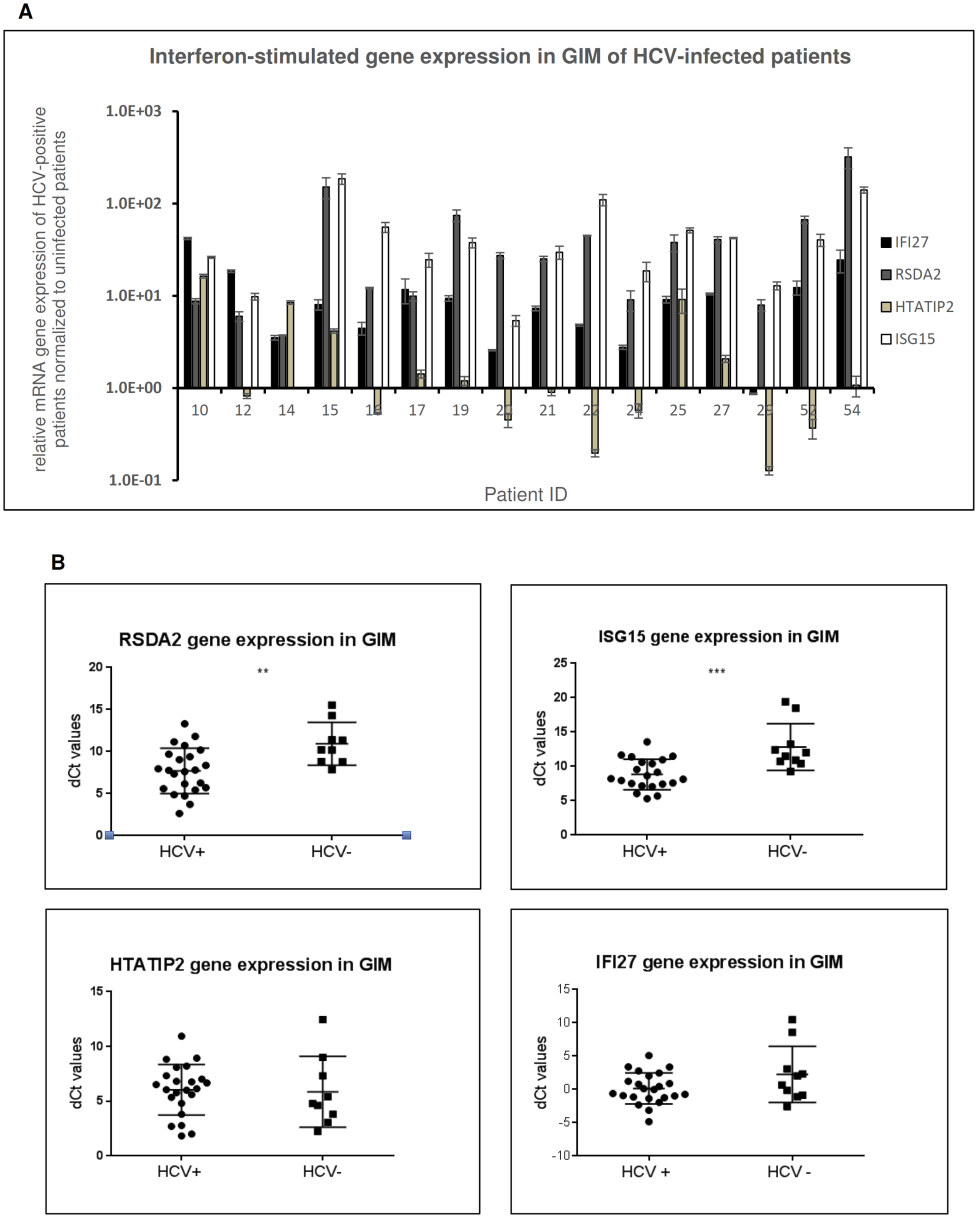
Semi-quantitative Real time PCR of IFN-regulated genes in HCV-positive patients GIM biopsies. Semi-quantitative Real time PCR of IFN-regulated genes: IFI27, RSAD2, ISG15 and HTATIP2 was performed in HCV-infected patients GIM biopsies. All samples have been analyzed in triplicate and normalized versus uninfected samples. The analysis showed high interferon-stimulated gene expression levels for most of the analyzed patients (A) and the ΔCt levels of HCV-positive patients (*n* = 16) resulted significantly higher compared to the ΔCt levels of the uninfected patients (*n* = 10) for RSAD2 (p<0.05) and ISG15 genes (p< 0.001) (B).

So far, we have found that enteroendocrine cells express receptors for HCV entry and that they sustain viral replication, as recapitulated by the *in vitro* data with NCI-H716 cells “Fig 6”. We were therefore puzzled by the histology of GIM sections that suggested that the titer of infection was quite low. As we observed that HCV could actively replicate within enteroendocrine cells as demonstrated by presence of HCV minus strand RNA, we questioned if host cells responded to HCV infection by mounting an anti-viral immune response to contain viral spread. Upon HCV infection, the innate immune system responds within days by inducing expression of hundreds of IFN stimulated genes (ISGs). In particular 4 genes, IFI27, RSAD2, ISG15 and HTATIP2, were previously identified by Dill et al. to be best the predictors of response to treatment with Interferon-alpha in a supervised classifier analysis of microarray data from liver biopsy specimens [44]. However, it is known that patients with their endogenous IFN system already activated before therapy with pegylated IFNα and ribavirin have a poor drug response. On the contrary, the same genes if induced in non-preactivated patients during treatment with pegylated IFNα and ribavirin are highly effective. In our study in order to establish whether the host mounts an IFN-mediated immune response during viral infection, we analysed expression of IFI27, RSAD2, ISG15 and HTATIP2 in GIM as described by Wieland et al. [45]. The results showed that high levels of ISGs were upregulated in gut biopsies normalized to the uninfected controls “Fig 7A”. In particular, the expression levels of IFI27, RSAD2 and ISG15 genes were high in most HCV-positive patients compared to uninfected control. Moreover, ΔCt values of RSAD2 and ISG15 were significantly higher in HCV-positive patients compared to the HCV-negative patients (p<0.05 and p< 0.001, respectively) “Fig 7B”. These data suggest that, after infection, cells of the GI mucosa responded by triggering an interferon-mediated inflammatory response. Despite our data demonstrated the presence of HCV in GIM compartment, the observed spread of infection was, however, very low in all patients, as confirmed by IHC. The high levels of expression of ISGs detected *in situ* can justify the reason of this limited viral diffusion in GIM compartment, as also demonstrated in the liver by Wieland et al. [45].

### HCV intra-tissue quasispecies variability

In order to evaluate the degree of HCV-HVR-1 variability in the different compartments, intra-tissue genetic distance and complexity (Shannon entropy) were calculated for the viral sequences obtained from pre-OLT tissues of each patient. The genetic distance was expressed as mean value of the genetic distance of the 10 clones obtained from each tissue of each patient.

In 3 of the 7 patients, intra-tissue genetic distance for GIM was lower than the one observed for liver and serum (patients 1, 4, 6; mean range of genetic distance 0.8–3.5%) with a complexity ranging from 0.27 to 0.55. In 3 of the 7 patients, intra-tissue GIM genetic distance and complexity were very high, (patients 2, 3, 7; mean range of genetic distance 32–42%, range of complexity 0.51–0.94) supporting the hypothesis that the virus can replicate in GIM and thus generate viral variants. For these patients intra-tissue GIM genetic distance was higher than that measured in the plasma, and higher than the one measured in the liver for 2 of the 3 patients. A low intra-tissue GIM GD was observed in 1 of the 7 patients (patient 5) with values very similar to the plasma (respectively 4.2% in GIM and 4% in plasma) and a complexity of 0.73 for the GIM and 0.87 for the plasma (Table 3). The median intra-patient genetic distances of HCV quasispecies from each compartment were: 4.2 in GIM, 4 in plasma and 28.4 in liver. Data comparison using Mann-Whitney test showed a significant difference in variability only between liver and plasma (p<0.05).

**Table 3:** Quasispecies analysis on pre-OLT tissues of each patient: Evaluation of Genetic distance, Entropy and dN/dS.

Compartment	ID patients	Genetic Distance (%)	Entropy	dS/dN
**Plasma**	**P1**	14.8 (range 0–33.3)	0.79	1.84
	**P2**	2.4 (range 0–5.3)	0.76	0
	**P3**	4.3 (range 0–11)	1	6.47
	**P4**	3.8 (range 0–8)	0.56	0
	**P5**	4 (range 0–11.3)	0.87	4.28
	**P6**	17.4 (range 1.2–37)	1	0.92
	**P7**	1.5 (range 0–6.4)	0.41	5.28
	**Median**	4	0.79	1.84
**GIM**	**P1**	3.5 (range 0–8.4)	0.57	7.23
	**P2**	37.8 (range 0–65.8)	0.51	1.06
	**P3**	32 (range 0–63)	0.94	1.66
	**P4**	0.8 (range 0–2.5)	0.4	1.28
	**P5**	4.2 (range 0–9.8)	0.73	41
	**P6**	1.4 (range 0–3.8)	0.52	0.62
	**P7**	41.7 (range 0–77)	0.53	1.43
	**Median**	4.2	0.53	1.43
**Liver**	**P1**	25.9 (range 0–48.5)	0.29	1.76
	**P2**	0.9 (range 0–2.6)	0.82	0
	**P3**	39.7 (range 0–86)	0.53	1.37
	**P4**	23.5 (range 0–70)	0.27	0.74
	**P5**	0 (only 3 sequences)	-	-
	**P6**	35.6 (range 0–72.6)	0.55	1.75
	**P7**	30.9 (range 0–67)	0.43	2.09
	**Median**	28.4	0.48	1.74

Median intra-patient entropy was 0.79 in plasma (range 0.41–1), 0.53 in GIM (range 0.4–0.94) and 0.48 in liver (range 0.29–0.82). Entropy was significantly higher in plasma compartment compared to liver (p<0.05).

To establish the presence of selective pressure on the mutation acquired from the virus we evaluated the dS/dN ratio. A selective pressure was found only in 1 patient in GIM (dS/dN = 0.62) and in 2 patients in plasma and in liver (dS/dN<1), likely because of the end-stage nature of the liver disease. Median intra-patient dS/dN values among each compartment were not significantly different (p<0.05). The values were: 1.84 in plasma (range 0–6.47), 1.43 in GIM (range 0.62–41), 1.74 in liver (range 0–2.09) (Table 3).

### Analysis of HCV compartmentalization

In order to evaluate the HCV GIM compartmentalization before transplantation, we compared the viral variants obtained from GIM with those from plasma and liver by quasispecies analysis of HCV-HVR1 region. Statistical approach and molecular phylogenesis were used to study tissue compartmentalization. Genetic distances obtained from sequences of each compartment for each patient were used to construct matrix for Mantel’s test analysis.

**Fig 8:**
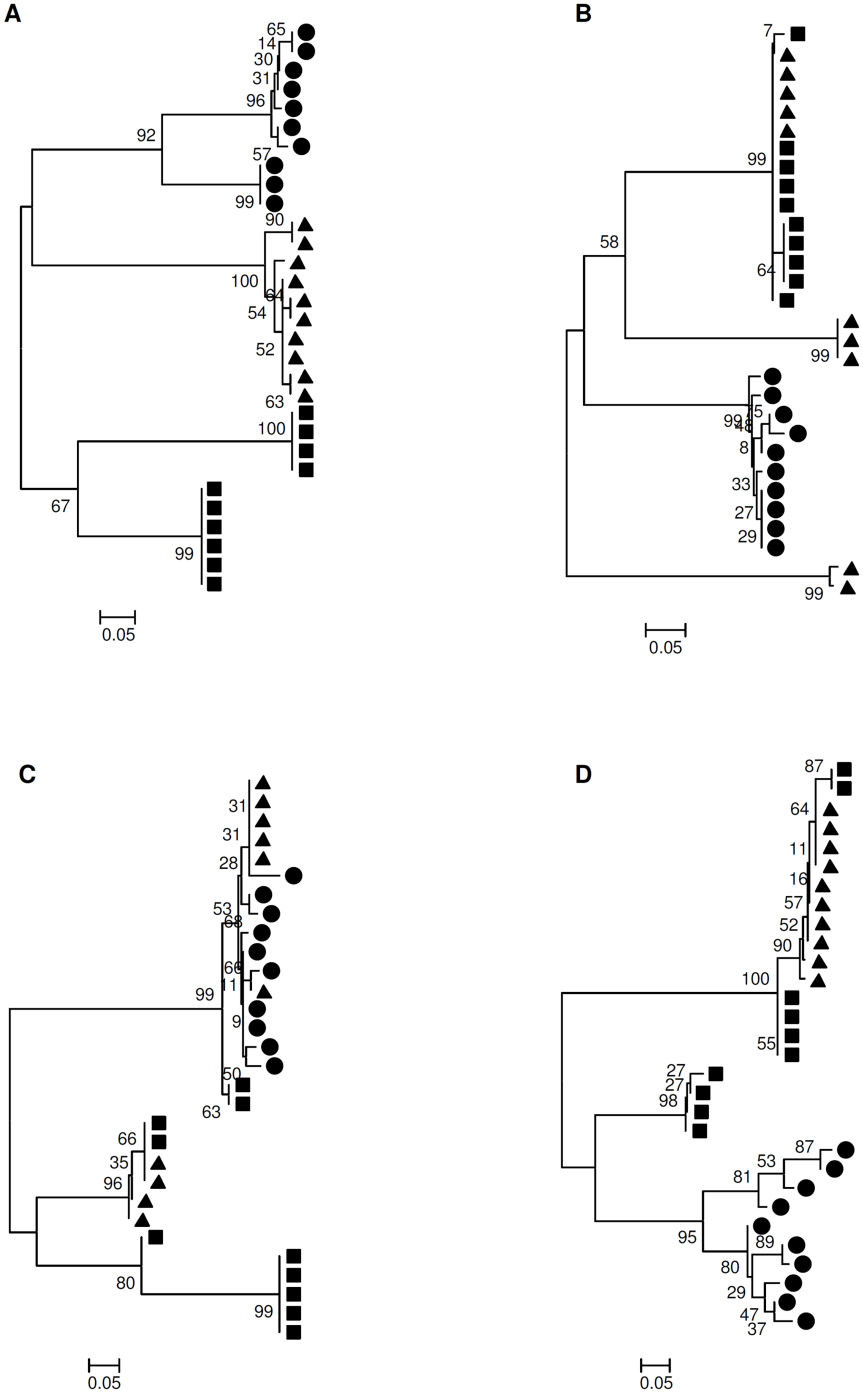
Phylogenetic trees of HVR1 regions of HCV sequences showing GIM HCV compartmentalization. Phylogenetic trees of HVR1 regions of HCV sequences using bootstrap values of 1000 replicates. The sequences from pre-transplantation plasma are showed by circles (●), GIM by triangles (▲) and liver by squares (■). (A-B) HCV compartmentalization of GIM vs liver and plasma (A- Patient 1) and of GIM vs plasma (B- Patient 2). Tree topology confirms Mantel’s test; (C-D) Partial HCV compartmentalization of GIM vs plasma (C- Patient 3) and of GIM vs liver (D- Patient 6).

By Mantel’s test we observed that HCV from GIM was compartmentalized compared with the plasma in 5 of the 7 patients (patients 1, 2, 4, 5, 6) and in 3 of the 7 patients compared with the liver (patients 1, 3, 4) (Table 4), suggesting an independent evolution of quasispecies in GIM compartment. These data were confirmed by bootstrapped phylogenetic trees, where it was evident that the GIM HCV sequences segregate independently compared with those isolated from other compartments. Phylogenetic analysis showed also a partial compartmentalization of sequences from the GIM in comparison with sequences from the liver in 3 patients (patients 2, 6, 7) and in comparison with sequences from the plasma in 2 patients (patients 3, 7) “Fig 8”. The overall analysis revealed that 6 out of 7 patients had pre-OLT HCV compartmentalization in sequences from GIM compared to the liver and that 7 out of 7 patients in sequences from GIM compared to the plasma.

**Table 4:** Genetic distances and Mantel’s test analysis of GIM compared to plasma and liver.

	GIM/plasma		GIM/liver	
Patient ID	GD(range)	Mantel’s test (p value)	GD (range)	Mantel’s test (p value)
**P1**	0.724 (0.660–0.859)	<0.0001^a^	0.690 (0.620–0.803)	<0.0001^a^
**P2**	0.546 (0.473–0.652)	<0.0001^a^	0.269 (0–0.649)	0.222
**P3**	0.277 (0.013–0.70)	0.066	0.536 (0–0.95)	= 0.0001^a^
**P4**	0.597 (0.55–0.70)	<0.0001^a^	0.643 (0.6–0.68)	<0.0001^a^
**P5**	0.615 (0–0.782)	<0.0001^a^	0.040^b^ (0–0.098)	-
**P6**	0.821 (0.720–0.994)	<0.0001^a^	0.291 (0.025–0.661)	0.085
**P7**	0.277 (0–0.82)	0.421	0.379 (0–0.81)	0.85

^a^ phylogenetic evidence of compartmentalization

^b^ analysis done on 3 clones

The genetic distance analysis performed on the sequences derived from pre-OLT tissues confirmed the Mantel’s test results, with high values in cases of compartmentalization, and lower values in cases of partial or no compartmentalization (Table 4).

### Phylogenetic analysis of HVR1

In order to evaluate the origin of HCV infecting newly transplanted liver, we compared the sequences of quasispecies herein present with that from other tissues such as GIM, liver and plasma pre-OLT and plasma post-OLT.

**Fig 9:**
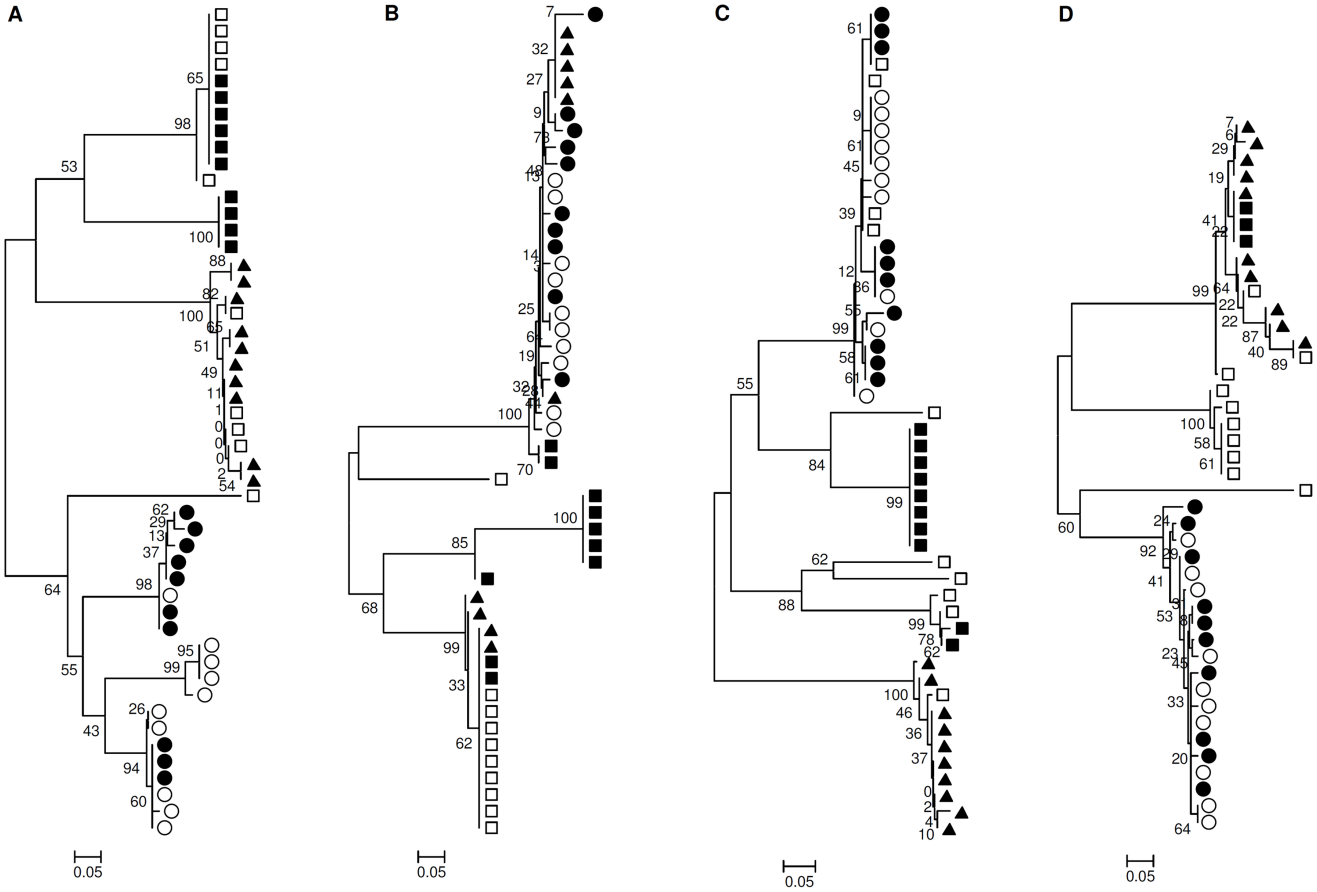
Phylogenetic trees of HVR1 regions of HCV sequences showing the quasispecies similarity between analyzed tissues. Phylogenetic trees of HVR1 regions of HCV sequences using bootstrap values of 1000 replicates. The sequences from pre-OLT plasma are showed by filled circles (●), GIM by filled triangles (▲) and liver by filled squares (■). The sequences from post-OLT plasma are showed by empty circles (○) and liver by empty squares (□). The bootstrap analysis showed that in most cases the HCV-HVR1 sequences derived from re-infected liver had great similarity with quasispecies from GIM and pre-OLT liver. (A) Patient 1 tree, (B) Patient 3 tree, (C) Patient 4 tree, (D) Patient 5 tree.

The phylogenetic tree analysis showed that in 4 out of 7 patients (1, 3, 4, 5) some viral sequences of HCV isolated from newly infected liver graft had a great sequence homology to the sequences derived from GIM, suggesting the hypothesis that, after transplantation, the virus produced in GIM homes the graft contributing to HCV relapse. In 4 patients we also found a sequence homology with pre-OLT liver sequences (patients 1, 3, 4, 5). In 2 patients some sequences were homologous with sequences from pre- and post-OLT plasma (patients 4, 6) “Fig 9”. In patient 7, most sequences derived from all tissues were very similar, with an inter-tissue genetic distance ranging from 0.004 to 0.4. Sequences from post-OLT liver were strongly associated with sequences from all tissues in the phylogenetic tree (intragroup genetic distance = 0.006). In patient 2, post-OLT liver quasispecies analysis was not done for a lack of biopsy samples. Any correlation was found between sequence homology and the time of samples collection.

## Discussion

It is accepted that HCV has a natural hepatic tropism, though several studies have demonstrated that the virus is present also in other compartments. However few are the reports on HCV infection and replication in extrahepatic tissues [6–11, 13, 14, 46–51]. In this study, we evaluated the role of the GIM compartment as a possible extrahepatic site for HCV replication and its contribution to newly-transplanted liver re-infection. Very few reports have considered the GIM as a site of HCV infection, and demonstrated the presence of viral RNA and antigens in this tissue [14, 46–50]. Yan and colleagues were able to detect HCV replication in intestine biopsy in only 1 out of 5 cases [14]. In our study, we found that the HCV is present and actively replicates in the GIM of chronically infected patients before and after liver transplantation. In particular, total HCV RNA was detected in 68% of the examined GIM biopsies collected before transplantation and in 76% of GIM biopsies collected after transplantation. HCV RNA minus strand was detected in 59% and 48% of the HCV RNA+ biopsies collected before and after transplantation, respectively. Moreover total HCV RNA was found also in 36% of not transplanted patients and HCV RNA minus strand was also detected in the 75% of the HCV RNA+ biopsies. The occurrence of virus in the GIM compartment is demonstrated also by the presence of viral antigens in gastrointestinal cells in 85% of patient's biopsies collected before transplantation, in 91% of patient's biopsies collected after transplantation, and in 73% of not transplanted patients. These data were confirmed also by the expression of the major HCV receptors by GIM cells. In particular, by co-staining we observed viral infection mainly in stromal and enteroendocrine cells. We confirmed that the virus can infect and replicate in the enteroendocrine cells using the neuroendocrine cell line NCI-H716. The enteroendocrine cells are scattered as single cells throughout the intestinal tract within the intestinal crypts and villi [38] and their function is to sense the luminal content and respond accordingly by secreting a variety of peptide hormones which control homeostatic and physiological functions in the digestive tract [52]. Enteroendocrine hormones are involved in the regulation of food intake, glucose homeostasis, energy consumption, intestinal peristalsis and lipid metabolism.

Accumulating evidence has highlighted a possible role of the gastrointestinal tract in HCV life cycle [47–50, 53, 54]. HCV circulating in the blood is strictly associated with very-low density lipoproteins (VLDL) and low-density lipoproteins (LDL) released by the liver during food metabolism [55–58]. In infected patients, circulating infectious particles are partly found in the low density fractions, associated with triacylglycerol (TG)-rich lipoproteins (TRL) denominated Lipo-Viro-Particles (LVP) and characterized by viral glycoproteins E1 and E2 on the surface and containing viral capsid and RNA [59]. Low density viral particles are of particular interest as they correlate with plasma infectivity, with lower density having increased infectivity. Intracellular HCV precursors have high density [60] and in the process of maturation in the host cells, HCV is transformed into low-density particles with assembly with Apolipoprotein-B (ApoB) molecules [57]. ApoB is a non exchangeable apolipoprotein which remains associated to the particle until its capture and internalization by lipoprotein receptors in the intestine. In humans, hepatocytes secrete very low density lipoproteins (VLDL) containing ApoB100, whereas enterocytes secrete another class of TRL, chylomicrons, which contain apoB48, the truncated form of apoB100 resulting from the enterocyte-specific editing. HCV envelope proteins are detected preferentially associated on the ApoB48 containing fraction, thus demonstrating the fundamental role of the intestine in HCV natural history [53]. Apo-B is a cellular factor essential for the assembly of infectious HCV particles [57]. HCV envelope glycoproteins have also the intrinsic capacity to utilize apoB synthesis and lipoprotein assembly machinery [54], thus raising the question of the contribution of the intestine to the viral load and suggesting that the virus could take advantage of TRL assembly and secretion for its own production and of TRL fate to be delivered to the liver. The presence of apoB48 in LVP supports the hypothesis of the existence of an intestinal site of HCV assembly and maturation for HCV [48, 53, 54].

During food processing, lipids are engulfed by GIM epithelial cells [48] that release in the blood chylomicrons carrying HCV particles [61]. As the intestine is connected to the liver via the enterohepatic circulation, these chylomicrons return to the hepatocytes where HCV particles are redelivered [10, 62]. As for ApoB48, Apo-E is another structural component of chylomicrons necessary for lipid uptake by the liver. Importantly, ApoE is found in association with HCV particles and it has been suggested that this complex might play a role in HCV entry in the host cells [63]. Recent work has demonstrated how ApoE is indispensable for the release of core protein from infected cells for infection spread. In the absence of ApoE, not only extracellular infectivity is abrogated, but also direct HCV cell-to-cell transmission is compromised. ApoE represents therefore as a host factor co-determining HCV tissue tropism [64]. Enteroendocrine cells are characterized by a rapid turnover with a lifespan of 4–6 days [52]. It is additionally possible that exfoliating infected cells might reach the liver but also other tissues and mediate cell-to-cell HCV transmission.

According to our data, the virus affects the function of enteroendocrine cells by inducing increased expression of somatostatin. Somatostatin, in fact, has an inhibitory effect on the immune system, inhibiting T cells proliferation and secretion of immunoglobulins and cytokines [39]. Also, HCV interaction with lipoproteins contribute to masking immunogenic viral glycoproteins from host immune system. These two events combined together might provide some hints to the mechanisms of viral escape from immunological pressure [65].

Since the emerging potential of enteroendocrine cells in intestinal regulation and immune system interaction during infective and inflammatory diseases, our data suggest to further investigate HCV interplay with these cells in the natural history of HCV infection. On the other hand, we have observed low viral replication rates in the GIM tissue. This limited viral diffusion in this compartment could be justified by the high levels of expression of ISGs observed. After viral infection, in fact, the host innate immune system induces the expression of hundreds of IFN stimulated genes (ISGs) in response to HCV. Despite this, in some infected patients HCV persists causing chronic infection during which other hundreds of type I and type III IFN stimulated genes are induced yet not resulting in viral eradication. HCV perhaps interferes with IFN signaling through the Jak-STAT pathway blocking the translation of ISG mRNAs [45]. The high level of expression of ISGs that we found in patient's biopsies may indicate the activation of immune system against the virus that may explain the low level of infection of GIM cells.

The presence of a quasispecies variability and positive values of Shannon entropy in GIM can confirm, furthermore, the ability of virus to replicate, but the very different values observed in the patients may suggest that other factors can contribute to HCV replication efficiency.

HCV compartmentalization in different tissues has been extensively reported [47, 48, 50, 62, 66], but, to the best of our knowledge, our study is the first to evaluate the existence of HCV compartmentalization in the GIM. Some support the idea that HCV undergoes compartmentalization under immunologic pressure with development of extrahepatic quasispecies that cannot infect the liver any longer [26]. Herein, quasispecies analysis of the HCV HVR1 region in all pre-OLT patients found a compartmentalization between the GIM and plasma, and in 6 out of 7 patients between the GIM and the liver. This result indicates that the virus can evolve independently in this tissue and that there is a possible involvement of the HCV particles present in the GIM in viral persistence and relapse of new-transplanted liver. Moreover, the phylogenetic analysis of sequences obtained from re-infected liver indicates no correlation between the observed sequence homology and the time of samples collection. Our data showed a different composition of HCV quasispecies of transplanted liver compared to quasispecies from plasma, highlighting that liver reinfection from blood is a rarer phenomenon, as supported by other studies [26, 67, 68]. Conversely, the bootstrap analysis showed that in most cases the HCV-HVR1 sequences derived from re-infected liver had great similarity with quasispecies from GIM and pre-OLT liver. The physiological connection between the GIM and the liver through the portal vein lends credence to the hypothesis that viral particles after replication in the GIM could gravitate directly to the liver. The observation that some GIM viral quasispecies were different from those in the serum suggests a direct viral-particle transfer from the GIM to the liver with no involvement of blood circulation. Moreover, the evidence of the molecular similarity between HCV variants in the re-infected liver and those present in GIM before transplantation proposes that HCV infection of GIM may play a role in virus persistence and reactivation.

Underpinning the implication of the intestine as a productive replicative extrahepatic reservoir would have relevant clinical implication in the prevention of liver reinfection after transplantation because anti-viral therapeutic aids targeting the GIM cells and their metabolism could be strategically designed to reduce the release of infectious virions by this compartment.

## Materials and methods

### Ethics statement

Samples were collected from each patient after written informed consent was obtained according to the protocol approved by the institutional research review board and ethics committee (Institutional Research Review Board of ISMETT 14/10). All adult subjects provided informed consent.

### Patients

The study was conducted from 2011 to 2016 by analyzing 97 gastrointestinal biopsies collected in Ismett between 2005 and 2014 (3 corpus, 38 antrum, 3 fundus, 8 antrum polyps, 1 fundus polyp, 6 colon, 1 sigmoid colon, 1 jejunum, 13 duodenum, 2 ileum, 1 cecum, 1 cecum polyp, 14 colon polyps, 1 sigmoid colon polyp, 3 rectum polyps, 1 duodenum polyp) from 76 patients: 11 HCV-negative patients and 65 HCV-positive patients, 54 of them HCV liver-transplanted patients, 11 HCV-positive patients suffering from other diseases (2 patients suffering from HCV-related hepatocellular carcinoma, 5 from kidney failure, 1 from gastritis, 1 from heart failure, 1 from cardiomyopathy and 1 from pulmonary emphysema). Among the 54 HCV liver-transplanted patients, of 29 we analyzed biopsies before and after transplantation. The patients were 59 males and 17 females of the same geographical area, with a mean age of 62.33 (range 22–75) years. All patients, but one, were positive for HCV RNAemia. In particular, out of the 29 patients with available biopsies before and after transplantation, 21 were HCV RNA positive before transplantation (16 patients showed a viral load higher than 700,000 IU/ml (range 700,000–100,000.000 IU/ml), 5 between 100,000 and 427,000 IU/ml (median: 165,500 IU/ml), 1 was negative and no data were available for the other 7 patients), and 27 after transplantation (22 patients showed a viral load higher than 700,000 IU/ml (range 842,000–2,750,000,000 IU/ml), 3 between 103,000 and 536,000 IU/ml (median: 427,000), one 4300 IU/ml, 1 was negative and no data were available for 1 patient) (Table 1). For the other 36 patients, 17 showed a viral load higher than 1,000,000 IU/ml (range 1,070,000–9,860,000), 5 between 500,000 and 1,000,000 (median: 617,000) and 14 lower than 500,000 IU/ml (range 55–416,400) (Table 2).

The majority of the patients were infected with HCV genotype 1b (53 of 65), 3 with 2a/2c, and 6 with 3a. For 3 patients no data were available. Twenty-five patients were treated with antiviral therapy against HCV, in particular, they received ribavirin in combination with α-interferon (IFN) or pegylated-IFN, no data were available for the other 40 patients. 11 HCV-negative patients were included in the study as negative controls (2 patients suffering from nephropathy, 4 from pulmonary fibrosis, 3 hepatic cirrhosis not related to HCV infection, 1 gastritis, and 1 colon cancer). For each patient, GIM biopsies and plasma samples were collected during routine evaluation visits, as planned for patients in waiting list for transplantation. For some patients biopsies pre- and post-OLT were available and for others also liver biopsies. The limited specimen availability allowed us to perform comparative analysis from plasma, GIM and liver samples taken before OLT, and plasma and liver taken after OLT only in 7 patients.

### HCV-RNAemia evaluation and HCV genotyping

HCV-RNA was extracted from plasma samples using High Pure System Viral Nucleic Acid kit (Roche Diagnostics, Manheim, Germany) following the manufacturer’s instructions and HCV RNA was amplified by Real-time PCR using COBAS Taq-Man HCV Test v2.0 (Roche Diagnostics) (range 25–3,91*10^8^ IU/ml) and COBAS TaqMan 48 Analyzer for automated amplification and detection. HCV genotype was determined by INNO-LipA HCV II kit (Innogenetics, Ghent, Belgium). Were indicated HCV-RNA was analyzed using COBAS Amplicor HCV Monitor test v.2.0 and COBAS Amplicor Analyzer (Roche Diagnostics) following the manufacturer’s instructions.

### HCV RNA detection in tissue samples by nested PCR

Total RNA was extracted from 5 sections of 5 μm thick paraffin-embedded GIM biopsies, using Nucleospin FFPE RNA (Macherey Nagel, Haerdt, France), and eluted in RNase-free water. RNA was retro-transcribed in cDNA and amplified using SuperScript III One-Step RT-PCR System with Platinum *Taq* DNA Polymerase (Invitrogen Corporation, Carlsbad, CA). Specific primers (0.2 μM each) for the 5’UTR region of HCV genome were used: HCV1 (5’-ACTCCACCATAGATCACTCC-3’) and HCV2 (5’-AACACTACTCGGCTAGCAGT-3’). An initial step at 50°C for 30’ was carried out to activate Superscript, followed by 2’ at 94°C and 35 cycles of PCR (15”at 94°C, 30” at 50°C, 30” at 72°C), with a final extension step at 72°C for 7’. Ten μl of the produced amplicons were subjected to a nested PCR in a volume of 50 μl containing 10X PCR Gold Buffer, 25mM MgCl_2_, dNTP 10mM, 1.25 U Taq Gold (Roche Molecular System Inc, Branchburg, New Jersey, USA), and 0.2 μM of primers HCV3 (5’-TTCACGCAGAAAGCGTCTAG-3’) and HCV4 (5’-GTTTATCCAAGAAAGGACCC-3’) [69].

The PCR cycle conditions were 94°C for 10’, 35 cycles at 94°C for 30”, 50°C for 30”, and 72°C for 30”, with a final extension at 72°C for 7’. The PCR products of 145 bp were analyzed with 2% agarose gel electrophoresis stained with Gel Red (Biotium, Hayward, CA).

### Strand specific RT-PCR for HCV RNA minus-strand detection in GIM biopsies

For the detection of negative-strand HCV RNA, total RNA obtained from paraffin-embedded GIM biopsies was retro-transcribed using the Reverse Transcription System (Promega, Madison, WI, USA) and 1.5 μM of the HCV1 sense primer. cDNA was then amplified by a first round PCR in a total volume of 50 μl containing 10X PCR Gold Buffer, 25mM MgCl_2_, dNTP 10mM, 1.25 U Taq Gold, and 0.2 μM each of HCV1 and HCV2 primer. PCR cycling condition were 94°C for 10’, 35 cycles of PCR (45”at 94°C, 45” at 50°C, 45” at 72°C) with a final extension at 72°C for 7’. Two μl of the first PCR product were subjected to a nested PCR using HCV3 and HCV4 primers. In order to generate a positive control of RNA minus-strand, the 5’UTR region of HCV genotype 1b, obtained from an HCV RNA-positive patient was amplified using the HCV1 and HCV2 primers, and was cloned in TOPO-TA vector (TOPO TA Cloning Kit Dual Promoter, Invitrogen, Carlsbad, CA), following the manufacturer’s instructions. The RNA-minus was synthesized, after assessing the insert orientation, by *in vitro* reverse transcription from the SP6 promoter of the recombinant plasmid, using the Riboprobe Combination System -SP6/T7 (Promega), and as described by Craggs et al. [70]. The sensitivity limit of this method was 10 genome equivalents; the strand specificity of the method was assessed using the positive and negative strand of HCV RNA as a template; the discrimination factor, between HCV RNA plus- and minus-strand detection, was 10^4^ fold (S2 and S3 Figs).

### Immunofluorescence and immunohistochemistry analysis

GIM formalin-fixed and paraffin-embedded biopsies were provided by ISMETT’s Pathology Laboratory. Sections of pre- and post-OLT GIM biopsies were deparaffinized and rehydratated using the Leica ST5020 Multistainer (Leica Microsystem, Wetzlar, Germany).

After washing with phosphate-buffered saline (PBS) (Lonza, Basel, Switzerland), heat-induced antigen retrieval step with citrate buffer (Dako, Carpinteria, CA, USA) was performed.

Blocking of non-specific sites was done using 3% bovine serum albumin (BSA) and 0.05% Tween 20 (both from Sigma-Aldrich, St. Louis, MO, USA) in PBS for 1–2 hours at room temperature.

Immunofluorescence assays were performed to detect the presence of the main HCV receptors, in particular the biopsy sections were incubated overnight in a humid chamber at 4°C with the following primary antibodies in blocking solution: rabbit polyclonal anti-SR-B1 (NB400-104, 1:200; C-terminal 450–509; Novus Biologicals, Cambridge, UK) [71] mouse monoclonal anti-CD81 (MON4103, 1:50; OCI-LY8; Uden, The Netherlands) [72], rabbit polyclonal anti-Claudin-1 (51–9000, 1:50; C-teminal; Invitrogen Corporation, Carlsbad, CA) [73], and rabbit polyclonal anti-Occludin (71–1500, 1:50; C-terminal 150 aa; Invitrogen Corporation, Carlsbad, CA) [74].

For the double staining analysis, in order to identify the specific virus infected-cells, mouse monoclonal anti-Chromogranin A (LK2H10, pre-dilute; secretory granules of most endocrine cells; Ventana, Tucson, AZ) [75] and goat polyclonal anti-core (2861, 1:5000; recombinant core; ViroStat Inc., Portland, ME) [76] were used as primary antibodies. After washes in PBS, the polyclonal fluorescein (FITC)-conjugated Affipure Donkey anti-goat and Cy3-conjugated Affipure Donkey anti-mouse (Alexa Fluor 488 and 594; Invitrogen Corporation, Carlsbad, CA) were used as secondary antibodies for 1 hours at room temperature.

The stained biopsy sections were mounted using SlowFade Gold Antifade reagent (Invitrogen Corporation, Carlsbad, CA) including 4,6-diamidino-2-phenylindole (DAPI) for the nuclear counterstaining and stored in the dark. Cell imaging was done with the fluorescence microscope Nikon Eclipse 50i (Nikon Corporation, Japan) coupled with a camera (Olympus XM10) and CellF software for image acquisition (Olympus).

For immunohistochemistry assay, performed to detect the presence of structural (E1, E2, Core) and nonstructural (NS3, NS4) HCV proteins in the GIM, after antigen retrieval, biopsy sections were incubated with methanol/3% H_2_O_2_ (both from Sigma-Aldrich) for 30 min to deactivate the endogenous peroxidase. Endogenous avidin and biotin were blocked using avidin/biotin blocking kit (Vector Laboratories, Inc.) and non-specific sites were blocked using normal serum of the Vectastain Elite ABC kit (Vector Laboratories Inc.) in PBS for 1–2 hours at room temperature.

The biopsy sections were incubated with the following primary antibodies overnight in a humid chamber at 4°C: mouse monoclonal anti-NS3 (MMM33, 1:50; Monosan, Uden, The Netherlands) [77]; mouse monoclonal anti-NS4 (5D4/10E7, 1:150; chimeric polyprotein of 90 aa; Santa Cruz Biotechnology, Inc., Santa Cruz, USA) [78]; mouse monoclonal anti-E1 (BDI198, 1:1000; Santa Cruz Biotechnology, Inc.) [78]; goat polyclonal anti-core (2861, 1:5000; recombinant core; ViroStat Inc., Portland, ME) [76] and goat monoclonal anti-E2 (ViroStat Inc.) [79]. The Vectastain Elite ABC kit and DAB peroxidase substrate kit (both from Vector Laboratories Inc.), were used as an antigen detection system following the manufacturer’s instructions. Sections were counterstained with Mayer’s Hematoxyline (Bio-Optica, Milano, Italy). Images were recorded with a Nikon digital camera (Nikon Corporation). The specificity of the immunohistochemical and immunofluorescence assays was tested by performing, for each antibody, a staining without the primary antibody “Figs 1, 3 and 4.”

### RT-PCR for gene expression analysis

Six GIM biopsies, 3 pre- and 3 post-OLT, from HCV-positive patients and 1 from a negative patients were collected for the analysis of the interferon stimulated genes IFI27, RSAD2, ISG15, HTATIP2. 15 GIM biopsies from HCV-positive patients and 2 from negative patients were collected for the analysis of somatostatin.

RNA was purified from all samples using Nucleospin FFPE RNA (Macherey Nagel). RNA was retro-transcribed in cDNA using the High Capacity cDNA Reverse Transcription kit (Applied Biosystems) in a total volume of 20 μl containing 10X RT Buffer, 10X RT random primers, 25X dNTP mix (100mM), Multiscribe Reverse Transcriptase 50U/ml, and RNAse inhibitor. The RT cycling conditions were 25°C for 10’, 37°C for 120’, and 85°C for 5’. The obtained cDNA was pre-amplified using the TaqMan PreAmp Master Mix kit (Applied Biosystems). In particular, cDNA, TaqMan preamp Master Mix 2X and each assay (IFI27, RSAD2, ISG15, HTATIP2, 18S, Chromogranin A, somatostatin and GAPDH) were mixed and pre-amplified at 90°C for 10’ followed by 10 cycles at 95°C for 15”, and 60°C for 4’. The pre-amplified cDNA was used to evaluate the expression of each gene by Realtime, using TaqMan Gene Expression Assay and Gene expression master Mix (Applied Biosystems). The experiment was performed in triplicate on the 7900 HT Fast Real Time PCR System (Applied Biosystem) according to the following cycle conditions: 95°C for 10’, 50 cycles at 95°C for 15”, and 60°C for 1’.

For gene expression analysis, a relative quantification was performed (2^-ΔΔCt^) using a housekeeping gene (18S for the analysis of interferon stimulated genes and GAPDH for the analysis of somatostatin) for the normalization and the data were compared to HCV negative patients as a calibrators.

### Transfection of Huh7.5 hepatic cell line with HCV JFH1 replicon

Plasmid pFK-Luc-Jc1 (GT 2a/2a) was a kind gift of Prof R Bartenschlager, Heidelberg University, Germany, with the authorization of Apath L.L.C., NY. The plasmid was linearized with restriction enzyme Mlu-I and transcribed into HCV RNA with MEGAScript^®^ T7 kit (Ambion). Permissive Huh7.5 cells (a kind gift of Prof R Bartenschlager, Heidelberg University, Germany, with the authorization of Apath L.L.C., NY), were transfected with HCV RNA by electroporation (3x10^6^ cells, 2,5μg RNA, buffer SE, program CA-138, Nucleofector 4D, Lonza). Efficiency of transfection was monitored by Luciferase activity with One-Glo Luciferase Assay System following manufacturer’s instructions (Promega). After addition of substrate, luminescence was quantified with Glomax 96 Microplate luminometer (Promega).

### Viral stock production

HCV viral stock were prepared as previously described [80]. Briefly, culture supernatants of HCV-infected Huh7.5 were clarified of cell debris by low-speed centrifugation (1,000 × g, 4°C, 10 min) and filtration through 0.45-μm-pore-size filter. The filtered culture supernatant was buffered with Hepes 20mM to stabilize pH to about 7.0 and then concentrated by the addition of 1/5 volume of ice-cold 40% PEG-8000/2.5M NaCl. HCV virus was precipitated at 4°C overnight, followed by centrifugation at 13000 × g at 4°C for 30 min. The precipitated virus was suspended and stored frozen at −70°C.

### Viral stock titration and immunostaining

12x10^3^/well Huh7.5 were seeded on 10mm diameter glass coverslip placed in 48-well plate.10-fold serially diluted viral stock was added to Huh7.5 cells in medium that was changed after 6 h. At 72 h post-infection, immunostaining against HCV core protein was performed. Briefly, cells were washed three times with PBS and fixed with ice-cold methanol at -20°C for 20 min. Cells were washed for three times with PBS, blocked for 1 h with PBS, 5%Normal Goat Serum (Biogenex, Fremont, CA, USA), 2% BSA and incubated with mouse anti-HCV Core (clone [C7-50], Abcam, Cambridge, UK) [81] diluted in blocking buffer 1:300 for an overnight at 4°C in a humidified chamber. Cells were washed with PBS and incubated for 1h at room temperature with secondary antibody goat anti-mouse IgG-Alexa568 (Invitrogen). The stained biopsy sections were mounted using SlowFade Gold Antifade reagent (Invitrogen) including DAPI for the nuclear counterstaining and stored in the dark. Images were acquired with confocal microscope (TCS SP5 II, Leica). The number of foci formed at the highest dilution was used to calculate the virus titer, which was expressed as the number of focus-forming units per milliliter of supernatant (FFU/ml). The titers of our JFH1 viral stock were usually in the range of 10^4^ to 10^6^ FFU/ml.

### NCI-H716 infection assay

Human NCI-H716 [H716] (ATCC^®^ CCL-251^™^) cells [82] were cultured in RPMI 1640 supplemented with 10% FBS, 2mM L-glutamine, 100 IU/ml penicillin and 100ug/ml streptomycin. Cells were grown in suspension at 37°C, in a humidified incubator in 5% CO_2_. NCI-H716 cells were seeded in 12-well plates at a density of 10^5^ cells per well and incubated for 24 hours at 37°C with serum-free medium containing HCV replicons at a MOI of infection as 0.02. 10^5^ Huh7.5 cells were inoculated with the virus as positive control of infection. Inoculum was removed and cells washed several times to remove excess of virus. Baseline virus concentration was determined (T0). Target cells were incubated in complete medium for 7 days and HCV concentration determined at day 1 (T1), day 2 (T2) and day 7 (T7) post-infection. The experiment was conducted in triplicate. HCV titer in the supernatant was quantified by Chemiluminescence Immune Assay (CMIA) technology using ARCHITECT HCV Ag assay (Abbott Laboratories, USA) following the manufacturer’s instructions. Infection was determined by immunofluorescence with HCV core mAb (clone [C-7-50], Abcam) as previously described on 20.000 cells spotted in glass slides by using Shandon cytospin centrifuge (Thermo Fisher Scientific). HCV viral load in the supernatant was evaluated by Real-time PCR using COBAS Taq-Man HCV Test v2.0 and COBAS TaqMan 48 Analyzer (Roche Diagnostics) at point T0, T1 and T7 days after the infection, in duplicate.

### HVR1 amplification and cloning

Total RNA was extracted from serum sample using the QIAamp MinElute Virus Spin kit (Qiagen GmbH, Hilden, Germany) and from GIM and liver biopsies, using the Nucleospin FFPE RNA kit (Macherey Nagel) and then transcribed using the Reverse Transcription System (Promega, Madison, WI, USA). cDNA was amplified by first round PCR in a total volume of 50 μl containing High Fidelity PCR Master Mix with HF Buffer (Thermo Scientific, Lithuania) and 0.2 μM of HVR1 and HVR2 primers, spanning the E1/E2 regions, including the entire HVR1 sequence. PCR conditions were 35 cycles at 98°C for 10”, 56°C for 20”, and 72°C for 20”, preceded by pre-heating at 98°C for 1’ and followed by a final extension at 72°C for 5’. PCR products were re-amplified in a second PCR using the primer pair HVR3/HVR4 with an annealing temperature of 58°C.

PCR templates were cloned in pGEM-T easy vector (pGEM-T Easy Vector Systems, Promega) using competent *E*.*coli* JM109 cells. Ten recombinant colonies for each sample were selected and incubated over night at 37°C in Luria Broth medium containing 50 μg/ml of ampicillin (Sigma-Aldrich Chemie, GmbH, Steinheim). Plasmid DNA from bacterial cultures was purified using the QIAprep Spin Miniprep kit (Qiagen GmbH) and restricted for 1 hour at 37°C using the EcoRI restriction enzyme (New England Biolabs, UK) to verify the presence of the insert. Plasmidic DNA was then labeled with the Big Dye terminator Cycle sequencing Kit v 3.1 (Applied Biosystems) and sequenced using ABI Prism 3500 Genetic Analyzer (Applied Biosystems).

Phylogenetic analysis was conducted on viral quasispecies obtained from patients’ samples before and after transplantation. The sequences were edited and HVR1 regions were aligned using ClustalW, integrated in BioEdit Software (Tom Hall, USA). Quasispecies analysis was performed using MEGA v 5.1 [83]. Intrapatient genetic distances were calculated by pairwise comparison of nucleotide sequences using Kimura’s two-parameter method [84] and were expressed as a percentage.

Synonymous (dS)/ non synonymous (dN) ratio was calculated as an index of selective pressure on viral quasispecies using the the Nei-Gojobori method with Jukes-Cantor correction [21, 85] Phylogenetic trees were constructed for each patient’s sequences with the neighbor-joining algorithm with 1,000 replicates of bootstrap sampling. Bootstrap values of ≥ 60% were considered significant [86, 87] Shannon entropy, which measures quasispecies complexity, was calculated at the nucleotide level using the following equation: -Σ(p_i_*lnp_i_)/lnN, where p_i_ is the relative frequency of each sequence in the viral quasispecies and N is the total number of analyzed sequence. The Shannon entropy values range from 0 to 1, where 0 means no complexity and 1 means highest complexity analysis [88].

Compartmentalization of HCV quasispecies was evaluated by molecular phylogenesis and by Mantel’s test as describes [26, 86, 89, 90]. Briefly, the Kimura two-parameter distance matrix of the sequences of each patient was compared with a similar matrix M (x, y) of the same size obtained by replacing distances with 0 if the sequences were from the same compartment and with 1 if the sequences were from distinct compartments. The Pearson correlation coefficient was computed using XLSTAT software with 10,000 permutations [26, 51, 88].

P values <0.05 were considered statistically significant, evidence that sequences from a given compartment were genetically closer to each other than to sequences from other compartments.

To avoid cross-contamination between samples, cloning assay of HCV HVR-1 sequences obtained from all tissues were performed in different times for each patients.

### Statistical analysis

Quantitative variables were expressed as means and standard deviations or medians and ranges, and categorical variables were expressed as absolute and relative frequencies. Data comparisons were performed with the paired Student’s t-test, Student’s t-test, Wilcoxon rank-sum test, Z test for proportion when were appropriate. Homoscedasticity and normal distribution assumptions of the t-tests were assessed with the Levene’s test and Shapiro-Wilk test. Statistical tests were considered significant with a corresponding P value <0.05. Data handling and statistical analyses were performed with Stata 13.0 and GraphPad Prism 6 software.

## Supporting information

S1 Fig IF assay on GIM biopsies of HCV-negative patients. Immunofluorescence assay on GIM biopsies of HCV-negative patients, using antibodies against HCV receptors: CD81, SR-B1, Claudin-1, Occludin (Original magnification X400). (A) CD81 in duodenum; (B) Claudin-1 in sigmoid colon; (C) Occludin in antrum; (D) SR-B1 in antrum. Scale bar: 50 μm.
(TIF)

S2 Fig Sensitivity limit of strand-specific RT-PCR for HCV minus strand detection. Sample dilutions were done to obtain 10.000, 5.000, 1.000, 100, 10 and 1 equivalent genomes of the HCV minus strand RNA. Dilutions were amplified using the RT-PCR protocol for minus strand HCV. The sensitivity of the method was 10 equivalent genomes of the HCV minus strand RNA.
(TIF)

S3 Fig Specificity of strand-specific RT-PCR for HCV minus strand detection. Dilutions of the plus strand HCV RNA were amplified using the RT-PCR protocol for minus strand HCV. The discrimination factor between plus and minus strand was of 10000 fold.
(TIF)

## References

[1] Scarselli E, Ansuini H, Cerino R, Roccasecca RM, Acali S, Filocamo G, et al The human scavenger receptor class B type I is a novel candidate receptor for the hepatitis C virus. EMBO J. 2002;21(19):5017–25. doi: 10.1093/emboj/cdf529 . 12356718 10.1093/emboj/cdf529 PMC129051

[2] Pileri P, Uematsu Y, Campagnoli S, Galli G, Falugi F, Petracca R, et al Binding of hepatitis C virus to CD81. Science. 1998;282(5390):938–41. . 9794763 10.1126/science.282.5390.938

[3] Evans MJ, von Hahn T, Tscherne DM, Syder AJ, Panis M, Wolk B, et al Claudin-1 is a hepatitis C virus co-receptor required for a late step in entry. Nature. 2007;446(7137):801–5. doi: 10.1038/nature05654 . 17325668 10.1038/nature05654

[4] Ploss A, Evans MJ, Gaysinskaya VA, Panis M, You H, de Jong YP, et al Human occludin is a hepatitis C virus entry factor required for infection of mouse cells. Nature. 2009;457(7231):882–6. doi: 10.1038/nature07684 . 19182773 10.1038/nature07684 PMC2762424

[5] Agnello V, Abel G, Elfahal M, Knight GB, Zhang QX. Hepatitis C virus and other flaviviridae viruses enter cells via low density lipoprotein receptor. Proc Natl Acad Sci U S A. 1999;96(22):12766–71. . 10535997 10.1073/pnas.96.22.12766 PMC23090

[6] Bare P. Hepatitis C virus and peripheral blood mononuclear cell reservoirs Patricia Bare. World J Hepatol. 2009;1(1):67–71. doi: 10.4254/wjh.v1.i1.67 . 21160967 10.4254/wjh.v1.i1.67 PMC2999261

[7] Bare P, Massud I, Parodi C, Belmonte L, Garcia G, Nebel MC, et al Continuous release of hepatitis C virus (HCV) by peripheral blood mononuclear cells and B-lymphoblastoid cell-line cultures derived from HCV-infected patients. J Gen Virol. 2005;86(Pt 6):1717–27. doi: 10.1099/vir.0.80882-0 . 15914850 10.1099/vir.0.80882-0

[8] Caussin-Schwemling C, Schmitt C, Stoll-Keller F. Study of the infection of human blood derived monocyte/macrophages with hepatitis C virus in vitro. J Med Virol. 2001;65(1):14–22. . 11505438 10.1002/jmv.1095

[9] Goutagny N, Fatmi A, De Ledinghen V, Penin F, Couzigou P, Inchauspe G, et al Evidence of viral replication in circulating dendritic cells during hepatitis C virus infection. J Infect Dis. 2003;187(12):1951–8. doi: 10.1086/375350 . 12792872 10.1086/375350

[10] Haruna Y, Kanda T, Honda M, Takao T, Hayashi N. Detection of hepatitis C virus in the bile and bile duct epithelial cells of hepatitis C virus-infected patients. Hepatology. 2001;33(4):977–80. doi: 10.1053/jhep.2001.23435 . 11283863 10.1053/jhep.2001.23435

[11] Januszkiewicz-Lewandowska D, Wysocki J, Pernak M, Nowicka K, Zawada M, Rembowska J, et al Presence of hepatitis C virus (HCV)-RNA in peripheral blood mononuclear cells in HCV serum negative patients during interferon and ribavirin therapy. Jpn J Infect Dis. 2007;60(1):29–32. . 17314422

[12] Moradpour D, Penin F, Rice CM. Replication of hepatitis C virus. Nat Rev Microbiol. 2007;5(6):453–63. doi: 10.1038/nrmicro1645 . 17487147 10.1038/nrmicro1645

[13] Sansonno D, Lauletta G, Montrone M, Tucci FA, Nisi L, Dammacco F. Virological analysis and phenotypic characterization of peripheral blood lymphocytes of hepatitis C virus-infected patients with and without mixed cryoglobulinaemia. Clin Exp Immunol. 2006;143(2):288–96. doi: 10.1111/j.1365-2249.2005.02987.x . 16412053 10.1111/j.1365-2249.2005.02987.x PMC1809584

[14] Yan FM, Chen AS, Hao F, Zhao XP, Gu CH, Zhao LB, et al Hepatitis C virus may infect extrahepatic tissues in patients with hepatitis C. World J Gastroenterol. 2000;6(6):805–11. doi: 10.3748/wjg.v6.i6.805 . 11819700 10.3748/wjg.v6.i6.805 PMC4728266

[15] Cacoub P, Comarmond C, Domont F, Savey L, Desbois AC, Saadoun D. Extrahepatic manifestations of chronic hepatitis C virus infection. Ther Adv Infect Dis. 2016;3(1):3–14. doi: 10.1177/2049936115585942 . 26862398 10.1177/2049936115585942 PMC4735500

[16] Grebely J, Matthews GV, Petoumenos K, Dore GJ. Spontaneous clearance and the beneficial impact of treatment on clearance during recent hepatitis C virus infection. J Viral Hepat. 17(12):896 doi: 10.1111/j.1365-2893.2009.01256.x . 20051007 10.1111/j.1365-2893.2009.01256.x

[17] Hajarizadeh B, Grebely J, Dore GJ. Epidemiology and natural history of HCV infection. Nat Rev Gastroenterol Hepatol. 2013;10(9):553–62. doi: 10.1038/nrgastro.2013.107 . 23817321 10.1038/nrgastro.2013.107

[18] Horner SM. Activation and evasion of antiviral innate immunity by hepatitis C virus. J Mol Biol. 2014;426(6):1198–209. doi: 10.1016/j.jmb.2013.10.032 . 24184198 10.1016/j.jmb.2013.10.032 PMC4431647

[19] Rosen HR. Emerging concepts in immunity to hepatitis C virus infection. J Clin Invest. 2013;123(10):4121–30. doi: 10.1172/JCI67714 . 24084744 10.1172/JCI67714 PMC3784533

[20] Demetris AJ. Evolution of hepatitis C virus in liver allografts. Liver Transpl. 2009;15 Suppl 2:S35–41. doi: 10.1002/lt.21890 . 19876940 10.1002/lt.21890

[21] Jackowiak P, Kuls K, Budzko L, Mania A, Figlerowicz M, Figlerowicz M. Phylogeny and molecular evolution of the hepatitis C virus. Infect Genet Evol. 2014;21:67–82. doi: 10.1016/j.meegid.2013.10.021 . 24200590 10.1016/j.meegid.2013.10.021

[22] Maasoumy B, Wedemeyer H. Natural history of acute and chronic hepatitis C. Best practice & research. 26(4):401–12. doi: 10.1016/j.bpg.2012.09.009 . 23199500 10.1016/j.bpg.2012.09.009

[23] Perz JF, Armstrong GL, Farrington LA, Hutin YJ, Bell BP. The contributions of hepatitis B virus and hepatitis C virus infections to cirrhosis and primary liver cancer worldwide. J Hepatol. 2006;45(4):529–38. doi: 10.1016/j.jhep.2006.05.013 . 16879891 10.1016/j.jhep.2006.05.013

[24] Plauzolles A, Lucas M, Gaudieri S. Hepatitis C virus adaptation to T-cell immune pressure. TheScientificWorldJournal. 2013;2013:673240 doi: 10.1155/2013/673240 . 23554569 10.1155/2013/673240 PMC3608127

[25] Powdrill MH, Tchesnokov EP, Kozak RA, Russell RS, Martin R, Svarovskaia ES, et al Contribution of a mutational bias in hepatitis C virus replication to the genetic barrier in the development of drug resistance. Proc Natl Acad Sci U S A. 108(51):20509–13. doi: 10.1073/pnas.1105797108 . 22135458 10.1073/pnas.1105797108 PMC3251051

[26] Ramirez S, Perez-Del-Pulgar S, Carrion JA, Costa J, Gonzalez P, Massaguer A, et al Hepatitis C virus compartmentalization and infection recurrence after liver transplantation. Am J Transplant. 2009;9(7):1591–601. doi: 10.1111/j.1600-6143.2009.02666.x . 19459796 10.1111/j.1600-6143.2009.02666.x

[27] Wyles DL. Antiviral resistance and the future landscape of hepatitis C virus infection therapy. J Infect Dis. 207 Suppl 1:S33–9. doi: 10.1093/infdis/jis761 . 23390303 10.1093/infdis/jis761

[28] European Association for the Study of the Liver. Electronic address eee. EASL Recommendations on Treatment of Hepatitis C 2016. J Hepatol. 2016. 10.1016/j.jhep.2022.10.006 36464532

[29] Negro F, Forton D, Craxi A, Sulkowski MS, Feld JJ, Manns MP. Extrahepatic morbidity and mortality of chronic hepatitis C. Gastroenterology. 2015;149(6):1345–60. doi: 10.1053/j.gastro.2015.08.035 . 26319013 10.1053/j.gastro.2015.08.035

[30] Lontok E, Harrington P, Howe A, Kieffer T, Lennerstrand J, Lenz O, et al Hepatitis C virus drug resistance-associated substitutions: State of the art summary. Hepatology. 2015;62(5):1623–32. doi: 10.1002/hep.27934 . 26095927 10.1002/hep.27934

[31] Reig M, Marino Z, Perello C, Inarrairaegui M, Ribeiro A, Lens S, et al Unexpected high rate of early tumor recurrence in patients with HCV-related HCC undergoing interferon-free therapy. J Hepatol. 2016;65(4):719–26. doi: 10.1016/j.jhep.2016.04.008 . 27084592 10.1016/j.jhep.2016.04.008

[32] Treatment of Recurrent HCV Infection following Liver Transplantation. Hepatitis C Online. 2016;Module 6: Treatment of Special Populations and Special Situations(Lesson 5):21.

[33] Elmasry S, Wadhwa S, Bang BR, Cook L, Chopra S, Kanel G, et al Detection of Occult Hepatitis C Virus Infection in Patients Who Achieved a Sustained Virologic Response to Direct-acting Antiviral Agents for Recurrent Infection After Liver Transplantation. Gastroenterology. 2016 . 27838287 10.1053/j.gastro.2016.11.002 PMC5285320

[34] Biggins SW, Bambha KM, Terrault NA, Inadomi J, Shiboski S, Dodge JL, et al Projected future increase in aging hepatitis C virus-infected liver transplant candidates: a potential effect of hepatocellular carcinoma. Liver Transpl. 18(12):1471–8. doi: 10.1002/lt.23551 . 23008049 10.1002/lt.23551 PMC3518670

[35] Ciesek S, Wedemeyer H. Immunosuppression, liver injury and post-transplant HCV recurrence. J Viral Hepat. 2012;19(1):1–8. doi: 10.1111/j.1365-2893.2011.01548.x . 22187942 10.1111/j.1365-2893.2011.01548.x

[36] Dhanasekaran R, Firpi RJ. Challenges of recurrent hepatitis C in the liver transplant patient. World J Gastroenterol. 2014;20(13):3391–400. doi: 10.3748/wjg.v20.i13.3391 . 24707122 10.3748/wjg.v20.i13.3391 PMC3974506

[37] Hiasa Y, Blackard JT, Lin W, Kamegaya Y, Horiike N, Onji M, et al Cell-based models of sustained, interferon-sensitive hepatitis C virus genotype 1 replication. Journal of virological methods. 2006;132(1–2):195–203. doi: 10.1016/j.jviromet.2005.10.014 . 16313977 10.1016/j.jviromet.2005.10.014 PMC2865175

[38] Sternini C, Anselmi L, Rozengurt E. Enteroendocrine cells: a site of 'taste' in gastrointestinal chemosensing. Curr Opin Endocrinol Diabetes Obes. 2008;15(1):73–8. . 18185066 10.1097/MED.0b013e3282f43a73 PMC2943060

[39] Gunawardene AR, Corfe BM, Staton CA. Classification and functions of enteroendocrine cells of the lower gastrointestinal tract. Int J Exp Pathol. 2011;92(4):219–31. doi: 10.1111/j.1365-2613.2011.00767.x . 21518048 10.1111/j.1365-2613.2011.00767.x PMC3144510

[40] Wakita T, Pietschmann T, Kato T, Date T, Miyamoto M, Zhao Z, et al Production of infectious hepatitis C virus in tissue culture from a cloned viral genome. Nat Med. 2005;11(7):791–6. doi: 10.1038/nm1268 . 15951748 10.1038/nm1268 PMC2918402

[41] Kato T, Date T, Murayama A, Morikawa K, Akazawa D, Wakita T. Cell culture and infection system for hepatitis C virus. Nat Protoc. 2006;1(5):2334–9. doi: 10.1038/nprot.2006.395 . 17406476 10.1038/nprot.2006.395

[42] Park JG, Kramer BS, Steinberg SM, Carmichael J, Collins JM, Minna JD, et al Chemosensitivity testing of human colorectal carcinoma cell lines using a tetrazolium-based colorimetric assay. Cancer research. 1987;47(22):5875–9. . 3664487

[43] Pfaender S, Brinkmann J, Todt D, Riebesehl N, Steinmann J, Steinmann J, et al Mechanisms of methods for hepatitis C virus inactivation. Appl Environ Microbiol. 2015;81(5):1616–21. doi: 10.1128/AEM.03580-14 . 25527548 10.1128/AEM.03580-14 PMC4325173

[44] Dill MT, Duong FH, Vogt JE, Bibert S, Bochud PY, Terracciano L, et al Interferon-induced gene expression is a stronger predictor of treatment response than IL28B genotype in patients with hepatitis C. Gastroenterology. 140(3):1021–31. doi: 10.1053/j.gastro.2010.11.039 . 21111740 10.1053/j.gastro.2010.11.039

[45] Wieland S, Makowska Z, Campana B, Calabrese D, Dill MT, Chung J, et al Simultaneous detection of hepatitis C virus and interferon stimulated gene expression in infected human liver. Hepatology. 2014;59(6):2121–30. doi: 10.1002/hep.26770 . 24122862 10.1002/hep.26770 PMC3975814

[46] Cheung DY, Kim JK, Kim JI, Han JY, Chung KW, Sun HS. [The detection of antigenic protein of HCV in gastric mucosa]. The Korean journal of gastroenterology = Taehan Sohwagi Hakhoe chi. 2005;45(4):294–300. . 15843755

[47] De Vita S, De Re V, Sansonno D, Sorrentino D, Corte RL, Pivetta B, et al Gastric mucosa as an additional extrahepatic localization of hepatitis C virus: viral detection in gastric low-grade lymphoma associated with autoimmune disease and in chronic gastritis. Hepatology. 2000;31(1):182–9. doi: 10.1002/hep.510310127 . 10613744 10.1002/hep.510310127

[48] Deforges S, Evlashev A, Perret M, Sodoyer M, Pouzol S, Scoazec JY, et al Expression of hepatitis C virus proteins in epithelial intestinal cells in vivo. J Gen Virol. 2004;85(Pt 9):2515–23. doi: 10.1099/vir.0.80071-0 . 15302945 10.1099/vir.0.80071-0 PMC2099454

[49] Miglioresi L, Riva E, Antonelli G, Russo F, Ricci GL. Localization of hepatitis C virus in gastrointestinal mucosa: a possible reservoir for relapse. Hepatology. 2003;38(3):775 doi: 10.1053/jhep.2003.50322 . 12939605 10.1053/jhep.2003.50322

[50] Tursi A, Brandimante G, Chiarelli F, Spagnoli A, Torello M. Detection of HCV RNA in gastric mucosa-associated lymphoid tissue by in situ hybridization: evidence of a new extrahepatic localization of HCV with increased risk of gastric malt lymphoma. Am J Gastroenterol. 2002;97(7):1802–6. doi: 10.1111/j.1572-0241.2002.05848.x . 12135039 10.1111/j.1572-0241.2002.05848.x

[51] Legendre P, Fortin MJ. Comparison of the Mantel test and alternative approaches for detecting complex multivariate relationships in the spatial analysis of genetic data. Molecular ecology resources. 10(5):831–44. doi: 10.1111/j.1755-0998.2010.02866.x . 21565094 10.1111/j.1755-0998.2010.02866.x

[52] Moran GW, Leslie FC, Levison SE, Worthington J, McLaughlin JT. Enteroendocrine cells: neglected players in gastrointestinal disorders? Therap Adv Gastroenterol. 2008;1(1):51–60. doi: 10.1177/1756283X08093943 . 21180514 10.1177/1756283X08093943 PMC3002486

[53] Diaz O, Delers F, Maynard M, Demignot S, Zoulim F, Chambaz J, et al Preferential association of Hepatitis C virus with apolipoprotein B48-containing lipoproteins. J Gen Virol. 2006;87(Pt 10):2983–91. doi: 10.1099/vir.0.82033-0 . 16963757 10.1099/vir.0.82033-0 PMC2043115

[54] Icard V, Diaz O, Scholtes C, Perrin-Cocon L, Ramiere C, Bartenschlager R, et al Secretion of hepatitis C virus envelope glycoproteins depends on assembly of apolipoprotein B positive lipoproteins. PLoS One. 2009;4(1):e4233 doi: 10.1371/journal.pone.0004233 . 19156195 10.1371/journal.pone.0004233 PMC2617766

[55] Merz A, Long G, Hiet MS, Brugger B, Chlanda P, Andre P, et al Biochemical and morphological properties of hepatitis C virus particles and determination of their lipidome. J Biol Chem. 2011;286(4):3018–32. doi: 10.1074/jbc.M110.175018 . 21056986 10.1074/jbc.M110.175018 PMC3024796

[56] Bridge SH, Sheridan DA, Felmlee DJ, Nielsen SU, Thomas HC, Taylor-Robinson SD, et al Insulin resistance and low-density apolipoprotein B-associated lipoviral particles in hepatitis C virus genotype 1 infection. Gut. 2011;60(5):680–7. doi: 10.1136/gut.2010.222133 . 20940286 10.1136/gut.2010.222133

[57] Gastaminza P, Cheng G, Wieland S, Zhong J, Liao W, Chisari FV. Cellular determinants of hepatitis C virus assembly, maturation, degradation, and secretion. J Virol. 2008;82(5):2120–9. doi: 10.1128/JVI.02053-07 . 18077707 10.1128/JVI.02053-07 PMC2258938

[58] Scholtes C, Ramiere C, Rainteau D, Perrin-Cocon L, Wolf C, Humbert L, et al High plasma level of nucleocapsid-free envelope glycoprotein-positive lipoproteins in hepatitis C patients. Hepatology. 2012;56(1):39–48. doi: 10.1002/hep.25628 . 22290760 10.1002/hep.25628

[59] Andre P, Komurian-Pradel F, Deforges S, Perret M, Berland JL, Sodoyer M, et al Characterization of low- and very-low-density hepatitis C virus RNA-containing particles. J Virol. 2002;76(14):6919–28. doi: 10.1128/JVI.76.14.6919-6928.2002 . 12072493 10.1128/JVI.76.14.6919-6928.2002 PMC136313

[60] Gastaminza P, Kapadia SB, Chisari FV. Differential biophysical properties of infectious intracellular and secreted hepatitis C virus particles. J Virol. 2006;80(22):11074–81. doi: 10.1128/JVI.01150-06 . 16956946 10.1128/JVI.01150-06 PMC1642172

[61] Hussain MM, Kancha RK, Zhou Z, Luchoomun J, Zu H, Bakillah A. Chylomicron assembly and catabolism: role of apolipoproteins and receptors. Biochim Biophys Acta. 1996;1300(3):151–70. . 8679680 10.1016/0005-2760(96)00041-0

[62] Mee CJ, Grove J, Harris HJ, Hu K, Balfe P, McKeating JA. Effect of cell polarization on hepatitis C virus entry. J Virol. 2008;82(1):461–70. doi: 10.1128/JVI.01894-07 . 17959672 10.1128/JVI.01894-07 PMC2224355

[63] Jiang J, Wu X, Tang H, Luo G. Apolipoprotein E mediates attachment of clinical hepatitis C virus to hepatocytes by binding to cell surface heparan sulfate proteoglycan receptors. PLoS One. 2013;8(7):e67982 doi: 10.1371/journal.pone.0067982 . 23844141 10.1371/journal.pone.0067982 PMC3699494

[64] Hueging K, Doepke M, Vieyres G, Bankwitz D, Frentzen A, Doerrbecker J, et al Apolipoprotein E codetermines tissue tropism of hepatitis C virus and is crucial for viral cell-to-cell transmission by contributing to a postenvelopment step of assembly. J Virol. 2014;88(3):1433–46. doi: 10.1128/JVI.01815-13 . 24173232 10.1128/JVI.01815-13 PMC3911621

[65] Catanese MT, Uryu K, Kopp M, Edwards TJ, Andrus L, Rice WJ, et al Ultrastructural analysis of hepatitis C virus particles. Proc Natl Acad Sci U S A. 2013;110(23):9505–10. doi: 10.1073/pnas.1307527110 . 23690609 10.1073/pnas.1307527110 PMC3677472

[66] Zeisel MB, Fafi-Kremer S, Fofana I, Barth H, Stoll-Keller F, Doffoel M, et al Neutralizing antibodies in hepatitis C virus infection. World J Gastroenterol. 2007;13(36):4824–30. doi: 10.3748/wjg.v13.i36.4824 . 17828813 10.3748/wjg.v13.i36.4824 PMC4611760

[67] Hughes MG Jr., Chong TW, Smith RL, Evans HL, Iezzoni JC, Sawyer RG, et al HCV infection of the transplanted liver: changing CD81 and HVR1 variants immediately after liver transplantation. Am J Transplant. 2005;5(10):2504–13. doi: 10.1111/j.1600-6143.2005.01060.x . 16162201 10.1111/j.1600-6143.2005.01060.x

[68] Hughes MG Jr., Rudy CK, Chong TW, Smith RL, Evans HL, Iezzoni JC, et al E2 quasispecies specificity of hepatitis C virus association with allografts immediately after liver transplantation. Liver Transpl. 2004;10(2):208–16. doi: 10.1002/lt.20060 . 14762858 10.1002/lt.20060

[69] Watanabe S, Kobayashi Y, Konishi M, Yokoi M, Kakehashi R, Kaito M, et al Appropriate interferon-alpha therapy for chronic hepatitis C: an assessment by quantitative changes in serum hepatitis C virus-RNA. Internal medicine (Tokyo, Japan). 1993;32(7):523–9. . 8286827 10.2169/internalmedicine.32.523

[70] Craggs JK, Ball JK, Thomson BJ, Irving WL, Grabowska AM. Development of a strand-specific RT-PCR based assay to detect the replicative form of hepatitis C virus RNA. Journal of virological methods. 2001;94(1–2):111–20. . 11337045 10.1016/s0166-0934(01)00281-6

[71] Koller E, Vincent TM, Chappell A, De S, Manoharan M, Bennett CF. Mechanisms of single-stranded phosphorothioate modified antisense oligonucleotide accumulation in hepatocytes. Nucleic Acids Res. 2011;39(11):4795–807. doi: 10.1093/nar/gkr089 . 21345934 10.1093/nar/gkr089 PMC3113586

[72] Guo M, Gong S, Maric S, Misulovin Z, Pack M, Mahnke K, et al A monoclonal antibody to the DEC-205 endocytosis receptor on human dendritic cells. Hum Immunol. 2000;61(8):729–38. . 10980384 10.1016/s0198-8859(00)00144-0

[73] Pfeiffer F, Schafer J, Lyck R, Makrides V, Brunner S, Schaeren-Wiemers N, et al Claudin-1 induced sealing of blood-brain barrier tight junctions ameliorates chronic experimental autoimmune encephalomyelitis. Acta Neuropathol. 2011;122(5):601–14. doi: 10.1007/s00401-011-0883-2 . 21983942 10.1007/s00401-011-0883-2 PMC3207130

[74] Furuse M, Itoh M, Hirase T, Nagafuchi A, Yonemura S, Tsukita S, et al Direct association of occludin with ZO-1 and its possible involvement in the localization of occludin at tight junctions. J Cell Biol. 1994;127(6 Pt 1):1617–26. . 7798316 10.1083/jcb.127.6.1617 PMC2120300

[75] Wagoner MP, Gunsalus KT, Schoenike B, Richardson AL, Friedl A, Roopra A. The transcription factor REST is lost in aggressive breast cancer. PLoS Genet. 2010;6(6):e1000979 doi: 10.1371/journal.pgen.1000979 . 20548947 10.1371/journal.pgen.1000979 PMC2883591

[76] Chen TC, Hsieh CH, Sarnow P. Supporting Role for GTPase Rab27a in Hepatitis C Virus RNA Replication through a Novel miR-122-Mediated Effect. PLoS Pathog. 2015;11(8):e1005116 doi: 10.1371/journal.ppat.1005116 . 26305877 10.1371/journal.ppat.1005116 PMC4549268

[77] Bassendine M, Bevitt D, Burt B. Detection of hepatitis C virus antigens in the recently transplanted liver of a chronically infected immunodeficient patient but not in biopsies of a native liver. The 11th International Congress of Virology, Sidney Australia. 1999:p. 227.

[78] Sansonno D, Lauletta G, Dammacco F. Detection and quantitation of HCV core protein in single hepatocytes by means of laser capture microdissection and enzyme-linked immunosorbent assay. J Viral Hepat. 2004;11(1):27–32. . 14738555 10.1046/j.1365-2893.2003.00474.x

[79] Lee C. Interaction of hepatitis C virus core protein with janus kinase is required for efficient production of infectious viruses. Biomol Ther (Seoul). 2013;21(2):97–106. doi: 10.4062/biomolther.2013.007 . 24009866 10.4062/biomolther.2013.007 PMC3762308

[80] Lindenbach BD, Evans MJ, Syder AJ, Wolk B, Tellinghuisen TL, Liu CC, et al Complete replication of hepatitis C virus in cell culture. Science. 2005;309(5734):623–6. doi: 10.1126/science.1114016 . 15947137 10.1126/science.1114016

[81] Arumugaswami V, Remenyi R, Kanagavel V, Sue EY, Ngoc Ho T, Liu C, et al High-resolution functional profiling of hepatitis C virus genome. PLoS Pathog. 2008;4(10):e1000182 doi: 10.1371/journal.ppat.1000182 . 18927624 10.1371/journal.ppat.1000182 PMC2564836

[82] Carmichael J, Park JG, Degraff WG, Gamson J, Gazdar AF, Mitchell JB. Radiation sensitivity and study of glutathione and related enzymes in human colorectal cancer cell lines. Eur J Cancer Clin Oncol. 1988;24(7):1219–24. . 2901354 10.1016/0277-5379(88)90131-9

[83] Tamura K, Peterson D, Peterson N, Stecher G, Nei M, Kumar S. MEGA5: molecular evolutionary genetics analysis using maximum likelihood, evolutionary distance, and maximum parsimony methods. Molecular biology and evolution. 2011;28(10):2731–9. doi: 10.1093/molbev/msr121 . 21546353 10.1093/molbev/msr121 PMC3203626

[84] Kimura M. A simple method for estimating evolutionary rates of base substitutions through comparative studies of nucleotide sequences. Journal of molecular evolution. 1980;16(2):111–20. . 7463489 10.1007/BF01731581

[85] Nei M, Gojobori T. Simple methods for estimating the numbers of synonymous and nonsynonymous nucleotide substitutions. Molecular biology and evolution. 1986;3(5):418–26. . 3444411 10.1093/oxfordjournals.molbev.a040410

[86] Ducoulombier D, Roque-Afonso AM, Di Liberto G, Penin F, Kara R, Richard Y, et al Frequent compartmentalization of hepatitis C virus variants in circulating B cells and monocytes. Hepatology. 2004;39(3):817–25. doi: 10.1002/hep.20087 . 14999702 10.1002/hep.20087

[87] Roque Afonso AM, Jiang J, Penin F, Tareau C, Samuel D, Petit MA, et al Nonrandom distribution of hepatitis C virus quasispecies in plasma and peripheral blood mononuclear cell subsets. J Virol. 1999;73(11):9213–21. . 10516029 10.1128/jvi.73.11.9213-9221.1999 PMC112955

[88] Blackard JT, Ma G, Welge JA, Martin CM, Sherman KE, Taylor LE, et al Analysis of a non-structural gene reveals evidence of possible hepatitis C virus (HCV) compartmentalization. J Med Virol. 2012;84(2):242–52. doi: 10.1002/jmv.22269 . 22170544 10.1002/jmv.22269 PMC3243959

[89] Schramm F, Soulier E, Royer C, Weitten T, Fafi-Kremer S, Brignon N, et al Frequent compartmentalization of hepatitis C virus with leukocyte-related amino acids in the setting of liver transplantation. J Infect Dis. 2008;198(11):1656–66. doi: 10.1086/592986 . 18925843 10.1086/592986

[90] Zehender G, De Maddalena C, Bernini F, Ebranati E, Monti G, Pioltelli P, et al Compartmentalization of hepatitis C virus quasispecies in blood mononuclear cells of patients with mixed cryoglobulinemic syndrome. J Virol. 2005;79(14):9145–56. doi: 10.1128/JVI.79.14.9145-9156.2005 . 15994809 10.1128/JVI.79.14.9145-9156.2005 PMC1168762

