# Supplementary figures and images for: HCV replication in gastrointestinal mucosa: Potential extra-hepatic viral reservoir and possible role in HCV infection recurrence after liver transplantation
Source: PLoS One. 2017 Jul 27;12(7):e0181683. doi: 10.1371/journal.pone.0181683 (PMC5531480; doi:10.1371/journal.pone.0181683)

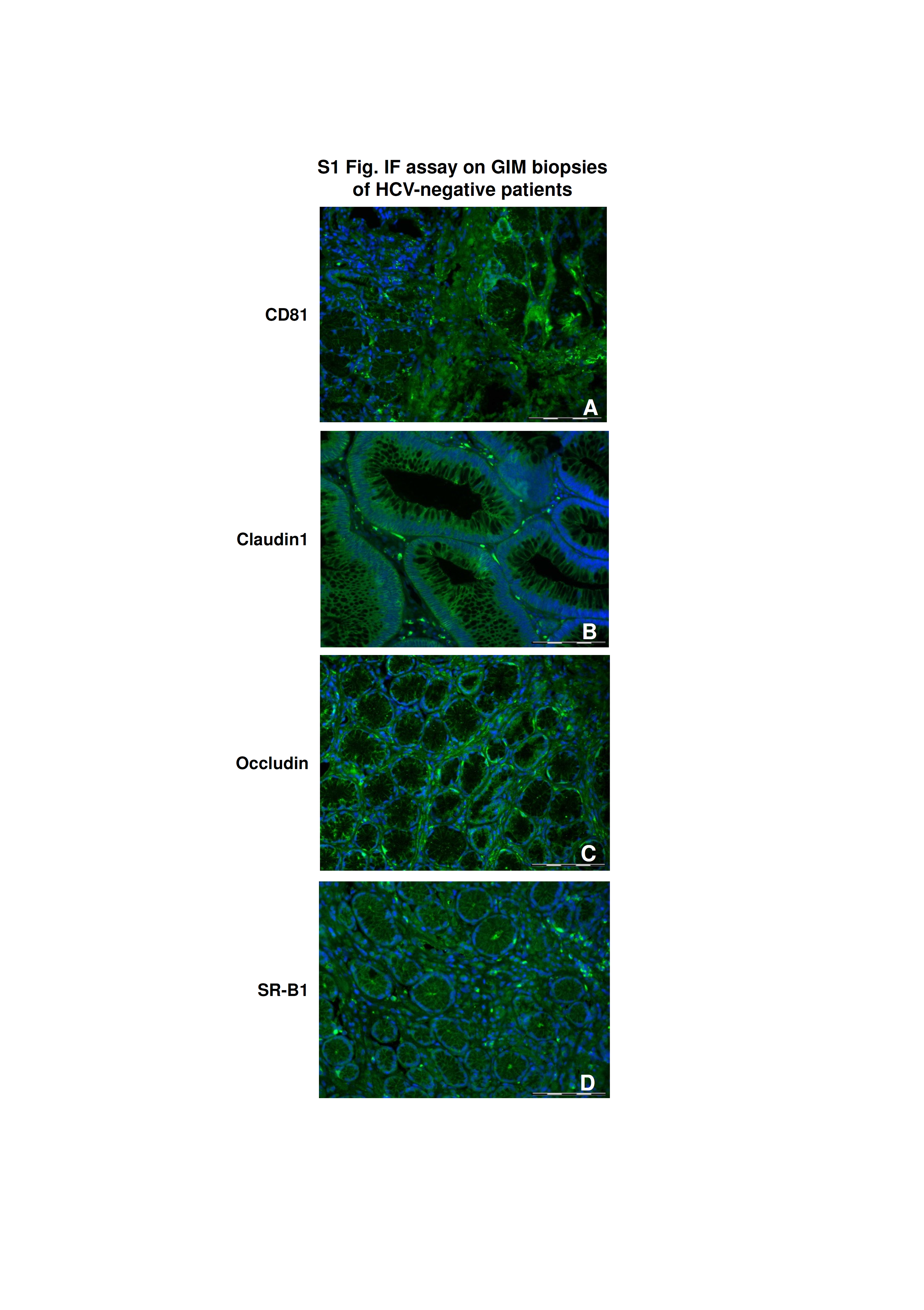

Supplement: S1 Fig — Immunofluorescence assay on GIM biopsies of HCV-negative patients, using antibodies against HCV receptors: CD81, SR-B1, Claudin-1, Occludin (Original magnification X400). (A) CD81 in duodenum; (B) Claudin-1 in sigmoid colon; (C) Occludin in antrum; (D) SR-B1 in antrum. Scale bar: 50 μm. (TIF) [file pone.0181683.s001.tif]

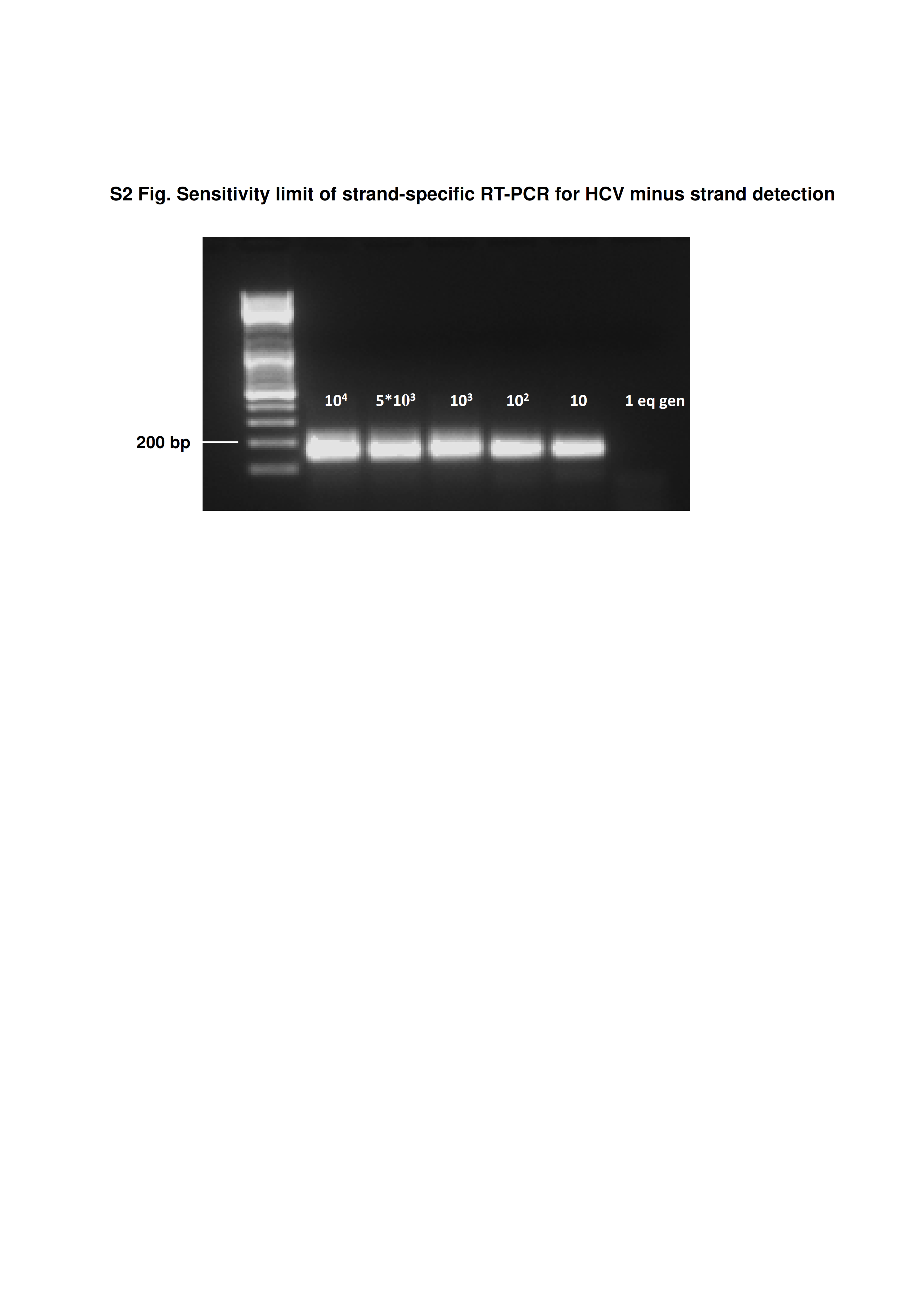

Supplement: S2 Fig — Sample dilutions were done to obtain 10.000, 5.000, 1.000, 100, 10 and 1 equivalent genomes of the HCV minus strand RNA. Dilutions were amplified using the RT-PCR protocol for minus strand HCV. The sensitivity of the method was 10 equivalent genomes of the HCV minus strand RNA. (TIF) [file pone.0181683.s002.tif]

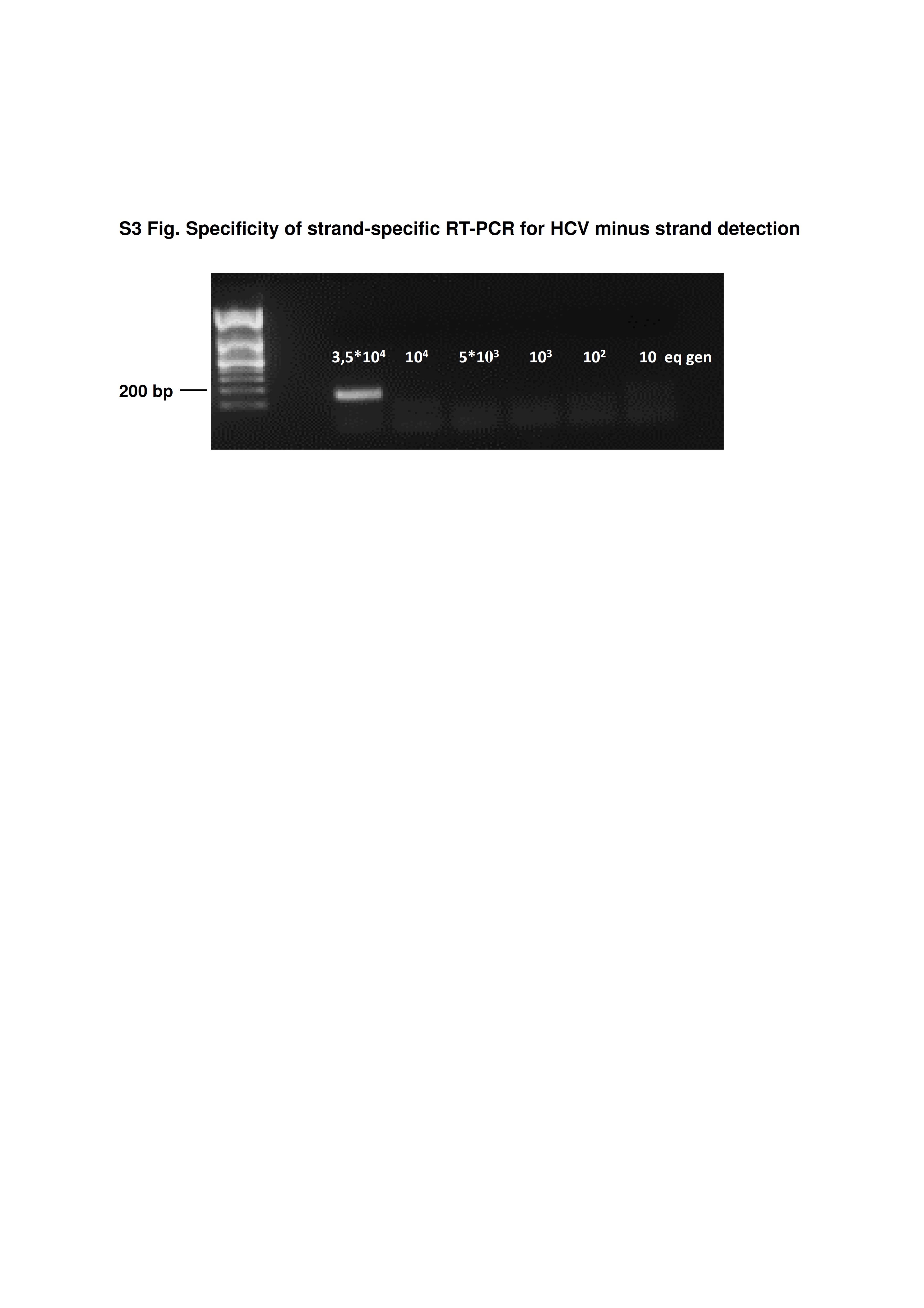

Supplement: S3 Fig — Dilutions of the plus strand HCV RNA were amplified using the RT-PCR protocol for minus strand HCV. The discrimination factor between plus and minus strand was of 10000 fold. (TIF) [file pone.0181683.s003.tif]
